# Small interfering RNA effect on lipoprotein(a): a systematic review

**DOI:** 10.1186/s43044-025-00635-1

**Published:** 2025-05-08

**Authors:** Omar Almaadawy, Mohamed Mar’ey Hesn, Yomna Ayman Elsalamony, Omar Ayman Abobakr, Abdelrahman Hossam Elshimy, Khaled Alsayed Abulkhair, Mahmoud Mohamed Negm, Ahmed Yasser Shaban, Yakubu Bene-Alhasan, Frank Annie, Adam Belcher, Ahmed Ramy Elashery

**Affiliations:** 1 Internal Medicine Department, MedStar Health, 201 E. University Pkwy, Baltimore, MD 21218 USA; 2 Faculty of Medicine, Al Azhar University, New Damietta, Egypt; 3 Faculty of Medicine, Portsaid University, PortSaid, Egypt; 4 Faculty of Medicine, Alexandria University, Alexandria, Egypt; 5 Faculty of Medicine, Zagazig University, Zagazig, Egypt; 6 Faculty of Medicine, Kafr Elsheikh University, Kafr Elsheikh, Egypt; 7 Cardiology Department, Charleston Area Medical Center, Charleston, WV USA

**Keywords:** siRNA, Lipoprotein(a), Lp(a), Inclisiran, Olpasiran, SLN360, Hyperlipidemia, Lipid-lowering therapy, Randomized controlled trials, LDL-c, Apolipoprotein B, Coronary artery disease, Cardiovascular risk

## Abstract

**Background:**

This systematic review investigates the effect of small interfering RNA (siRNA) therapies on lipoprotein(a) [Lp(a)] levels. The purpose is to evaluate the outcomes of recent randomized controlled trials (RCTs) involving siRNA treatments aimed at lowering Lp(a) levels, a known cardiovascular risk factor.

**Methods:**

A comprehensive search across multiple databases was conducted, identifying 20 published and ongoing RCTs that examined the effects of siRNA therapies such as inclisiran, olpasiran, and SLN360 on Lp(a) levels. The included studies were analyzed to assess Lp(a) reductions and other lipid-related outcomes.

**Results:**

The RCTs demonstrated significant reductions in Lp(a) levels following siRNA therapy. Additional reductions were noted in LDL-c and apolipoprotein B levels. Side effects were typically mild, including injection site reactions.

**Conclusions:**

siRNA therapies show promise in effectively lowering Lp(a) levels, with minimal adverse effects. However, further research is required to establish their long-term safety and efficacy.

**Supplementary Information:**

The online version contains supplementary material available at 10.1186/s43044-025-00635-1.

## Highlights

1. The review evaluates the efficacy of SiRNA therapies, including inclisiran, SLN360, and olpasiran, in effectively lowering Lp(a) and LDL-C levels, which are critical in reducing the risk of cardiovascular diseases.
2. Olpasiran is more effective in lowering Lp(a) more than inclisiran and SLN360, while inclisiran is more effective in lowering LDL-C more than olpasiran and SLN360.
3. It assesses the safety profiles of these SiRNA drugs, finding them generally favorable across the studies analyzed, with injection site pain being the most reported side effect. Long-term safety profiles yet to be determined.
4. The analysis spans various SiRNA medications, providing a comparative insight into their performance, dose-responsiveness, and the potential for personalized treatment approaches in lipid management.
5. The call for further research is emphasized, particularly the need for more extensive and rigorous randomized controlled trials. These future studies are crucial for validating the long-term benefits, safety, and efficacy of SiRNA therapies in a diverse patient demographic.
6. Additionally, there is advocacy for studies focusing specifically on the reduction of cardiac risk factors. Understanding SiRNA therapies' impact on cardiovascular risk factors beyond Lp(a) and LDL-C could revolutionize preventive strategies in cardiovascular health.

**Supplementary Information:**

The online version contains supplementary material available at 10.1186/s43044-025-00635-1.

## Background

Coronary artery disease (CAD) has been on the rise and remains the leading cause of death globally [1]. Nevertheless, its onset can be mitigated through modifying various risk factors, notably dyslipidemia and diabetes mellitus [1]. Low-density lipoprotein cholesterol (LDL-C) has been extensively studied as an established risk factor for CAD; hence, cholesterol reduction has been a primary intervention in preventing CAD [2]. Current guidelines and randomized controlled trials (RCTs) have shown that reducing LDL-C levels to below 70 mg/dl is advantageous for high-risk patients, with new guidelines from the European Society of Cardiology even advising reducing LDL further to < 55 mg/dl [3, 4]. However, other lipoproteins may increase the risk of CAD and should be addressed, such as lipoprotein(a) (Lp(a)), a molecule bound to apolipoprotein(a) [5] with about one-fifth of the world's population (approximately 1.4 billion individuals) have high Lp(a) levels [5, 6]. Lp(a) is considered proatherogenic, proinflammatory, and prothrombotic, with cumulative data suggesting a causal relationship with atherosclerotic cardiovascular disease (ASCVD) [7]; CAD risk has been associated with both high Lp(a) molar concentration and apo(a) size, which are inversely correlated properties of the Lp(a) particle that is mostly genetically determined and highly heterogeneous in the general population [8]. Several national and international societies such as the European Society of Cardiology/European Atherosclerosis Society recommend Lp(a) testing if an individual has documented ASCVD (especially with recurrent events on optimal lipid-lowering therapy), severe hypercholesterolemia or genetic familial hypercholesterolemia (FH), premature ASCVD, or a first-degree family member with premature ASCVD, particularly in the absence of traditional risk factors [9]. Clinicians predominantly utilize statins to lower LDL-C. Statins inhibit 3-hydroxy-3-methyl-glutaryl-coenzyme A (HMG-CoA) reductase—the critical step in hepatic cholesterol synthesis [10]. If the response to statins is suboptimal, ezetimibe can be added which impedes the intestinal NPC1-like intracellular cholesterol transporter 1, reducing dietary cholesterol absorption [10]. Studies have indicated that when combined with statins, ezetimibe can potentially lower the risk of death from cardiac diseases [10, 11]. However, some patients may not achieve optimal control, necessitating additional treatments such as bempedoic acid (which works by inhibiting the adenosine triphosphate-citrate lyase, an early step in the cholesterol synthesis pathway) and a range of emerging small molecular approaches [10]. Despite its significant role in cardiovascular risk, Lp(a) remains difficult to manage because current therapies, including statins, ezetimibe, and bempedoic acid, have minimal or no impact on lowering Lp(a) levels [12]. siRNA addresses this gap by targeting Lp(a) at the gene expression level, offering a promising approach beyond the reach of existing therapies [13]. In the context of comparison, inclisiran, a Proprotein convertase subtilisin/kexin type 9 (PCSK9, which reduces the number of LDL receptors on the surface of hepatocytes by enhancing their degradation) siRNA inhibitor—lower PCSK9 and prevent LDL receptors degradation—offers a less frequent dosing schedule every 3–6 months, making it more convenient compared to daily statins [14]. In addition to this, inclisiran maintains higher LDL-C reductions, as much as a mean 55% reduction in LDL levels at 6 months of treatment, compared with a 40% reduction from statins alone, and in combination with statins, the combination of atorvastatin and inclisiran was associated with a reduction in LDL-C of as much as 65%, compared with a reduction of as much as 40% with atorvastatin alone [15].

**Fig. 1:**
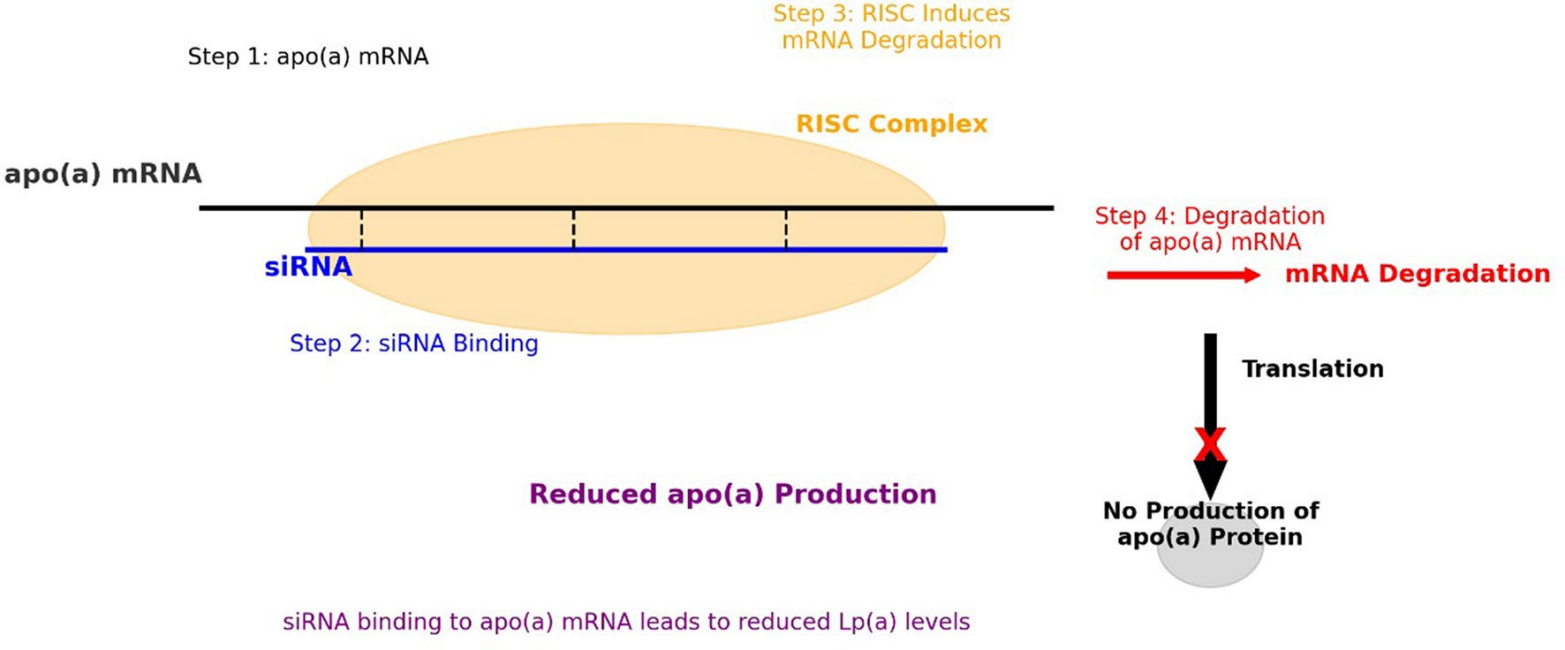
Small interfering RNA (siRNA) mechanis of action

Regarding Lp(a) reduction, past and current trials have been investigating the effectiveness of siRNA and antisense oligonucleotide (ASO) [5]. siRNA was first identified in 1998 by Fire and Mello, a discovery that deepened our grasp of gene regulation [13]. siRNA offers a novel approach by targeting gene expression directly, thus addressing elevated Lp(a) levels at the molecular level. siRNA molecules are designed to bind to complementary sequences on messenger RNA (mRNA), which is responsible for translating specific genes into proteins. In the case of Lp(a), siRNA targets the mRNA encoding apolipoprotein(a), the unique protein component of Lp(a) particles that contribute to its properties. Once bound to apo(a) mRNA, siRNA triggers a cellular mechanism called RNA-induced silencing complex (RISC), which leads to mRNA degradation and effectively silences the gene responsible for apo(a) production (Fig. 1) [10, 13, 16]. Inclisiran was the inaugural siRNA approved for treating familial hyperlipidemia, inhibiting PCSK-9 synthesis—a factor in LDL-C receptor degradation [10]. Ongoing studies on other siRNAs, like olpasiran, which targets apo(a) synthesis, are underway, with some being in phase II. Additional siRNA therapies include SLN360 and lepodisiran, which also targets apo(a) synthesis [17, 18]. This study systematically reviews the latest RCTS and literature to examine various SIRNA therapies, including the effect of SIRNA on LP(a), and to understand its potential in shaping the future landscape of hyperlipidemia treatment and addressing the gap in the hyperlipidemia treatment and reducing the risk of CAD.

## Methods

### Search strategy

**Fig. 2:**
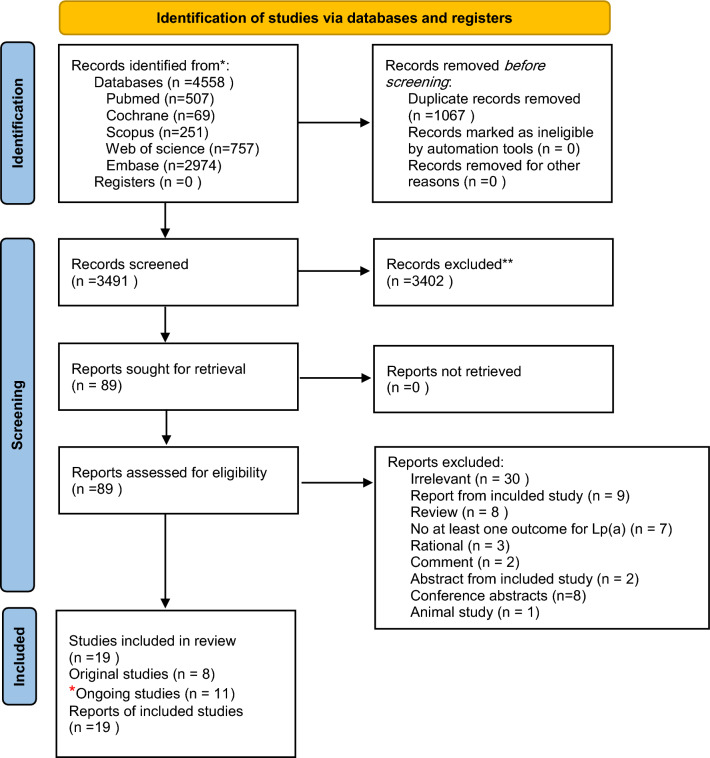
PRISMA 2020 flow diagram for new systematic reviews which included searches of databases and registers only. Red color * indicates, ongoing studies identified manually (*n* = 11). Black color * consider, if feasible to do so, reporting the number of records identified from each database or register searched (rather than the total number across all databases/registers). Black color ** indicates, if automation tools were used, indicate how many records were excluded by a human and how many were excluded by automation tools

We reported this systematic review following the preferred reporting items for systematic review (PRISMA) (Fig. 2) [19]. We systematically searched databases, including PubMed, Scopus, Web of Science, Cochrane, and Embase, using the search terms Intervention (small interfering RNA) and Outcome (lipoprotein-a). Relevant keywords and Medical Subject Headings (MeSH) terms were retrieved and incorporated into an appropriate search strategy. We imported initial search results in Rayyan for title and abstract screening after duplicates removal using endnote version x8.0.1. Six authors screened all included studies according to our eligibility criteria by title and abstract. Any relevant studies and conflict studies were shifted to full text screening. We used Blind mode in reviewing to avoid any possible bias, each paper has been reviewed by two authors blindly then conflicts have been resolved at the end by the lead author. The search was performed in December 2023. No language, study design restrictions, or filters were used during the search. Figures were created using the R programming language [20].

### Inclusion criteria

We included studies that met these inclusion criteria: (1) primary studies that used SiRNA preparations and had a comparison and control group, (2) assessing safety and efficacy and reporting clear outcomes related to Lp(a) level or concentration. We excluded secondary articles, including reviews and meta-analyses, preclinical studies, animal studies, gray literature, conference abstracts, abstracts from included studies and pharmacokinetics and pharmacodynamics studies, which had no efficacy or safety outcomes, to maintain the validity of our review. We also did not extract data from studies that fit our inclusion criteria but were ongoing. At least two independent authors reviewed each study, and any disagreement was resolved by discussion between the two authors and a senior author’s decision. The full text of all eligible articles was then obtained and checked by at least two independent authors.

### Data extraction and risk of bias assessment

At least two independent authors extracted data from each study using a pre-formed Excel book. The extracted data were: (1) details of the study including (author name, year, study design, duration, patients or disease, drug use, study arms, inclusion criteria, exclusion criteria, primary outcome, secondary outcome, and conclusion); (2) baseline characteristics of participants in the study, including (study ID, intervention name, age, gender, race, body mass index, cardiovascular risk factors, smoking, diabetes mellitus, and familial hypercholesterolemia); (3) participant baseline lipid profile and therapies; (4) safety outcomes including (adverse events, injection site reaction, myalgias, liver-related adverse events, kidney-related adverse effects, thrombocytopenia, hyperglycemia, and others); (5) efficacy outcomes including (the maximum absolute change in Lp(a), maximum mean percent change from baseline of Lp(a), Lp(a) mean percent change from baseline, mean percent change from baseline in corrected LDL-c, mean percent change from baseline in ApoB, mean percent change from baseline in high-density lipoprotein cholesterol (HDL-C), and mean percent change from baseline in triglycerides).

At least two independent authors reviewed each study protocol, full text, and available supplementary material for risk of bias assessment. The Cochrane risk-of-bias assessment tool of randomized trials—ROB-2—recommended by the Cochrane Handbook for systemic reviews, was used to assess RCTs [21]. The ROB-2 tool includes five domains for risk of bias assessment: (1) randomization process, (2) deviation from intended intervention, (3) missing outcome data, (4) measurement of the outcome, (5) selection of the reported results.

## Results

### Study selection and characteristics

The initial search identified 3711 studies (Supplementary Table 7). Of these studies, 3605 were excluded, and the remaining 106 were screened by reading full texts (Fig. 2). Eventually, we identified 20 RCTs, nine completed and eleven still ongoing (Supplementary Table 5) [22–40], that assessed the effect of siRNA therapies in LP(a) reduction. The nine completed RCTs had various interventions, including inclisiran, olpasiran, SLN 360, and lepodisiran [18, 22–29]. Publication dates ranged from 2017 to 2023. The follow-up periods ranged from 150 to 540 days. Among these studies, adults with Lp(a) level more than 150 mmol/l [22, 23, 31, 38] or patients with LDL-C more than 100 mg/dl in the absence of cardiovascular diseases [24–26, 29] were deemed eligible to be included, while the usage of any monoclonal antibody drug targeting PCSK9 was considered as an exclusion criterion [24–27, 36, 37, 39, 40] (Supplementary Tables 4 and 5). 

### Quality assessment

We evaluated the methodological quality of the nine studies in our review using various tools specific to each study’s design, as mentioned above in the methods. Studies were stratified as low risk of bias, high risk of bias, or some concerns, based on prespecified criteria in the ROB-2 tool [21]; two studies [28, 29] were considered to be high risk of bias, whereas six studies [18, 22–26] were considered to be low risk of bias. One study was considered to have some concerns [27] (Supplementary Tables 6 and 7).

### Characteristics of patients in included studies

The mean age of the participants varied between 42 and 67 [22–29]. Six studies included patients with cardiovascular risk factors, including hypertension [18, 22–27], diabetes mellitus [22, 24–27], positive smoking status [24–27], and familial hypercholesterolemia [22, 24, 26] (Table 1 and supplementary Table 4). The other studies excluded participants with cardiovascular risk factors [28, 29].

**Table 1:** Baseline characteristics of the included studies

References	Intervention name	Number of individuals (n.)	Age (mean/SD)	Gender Male (N%)	Race			Body mass index (BMI) (Mean/SD)	Cardiovascular risk factors			
					White (N%)	Black (N%)	Others (N%)		Hypertension (N%)	Diabetes (N%)	Smoking (N%)	Familial hypercholesterolemia (N%)
Fitzgerald et al. [29]	Placebo single dose	6	48 (14.2)	2 (33.3%)	4 (66.7%)	2 (33.3%)	0 (0%) (Asian)	24.9 (3.17)	–	–	–	–
	Inclisiran 25 mg single	3	47 (14.2)	3 (100%)	2 (66.7%)	1 (33.3%)	0 (0%)	27.7 (0.21)	–	–	–	–
	Inclisiran 100 single dose	3	48 (6.2)	3 (100%)	3 (100%)	0 (0%)	0 (0%)	25.5 (2.10)	–	–	–	–
	Inclisiran 300 single dose	3	48 (12.7)	3 (100%)	1 (33.3%)	1 (33.3%)	1 (33.3%)	27.0 (1.29)	–	–	–	–
	Inclisiran 500 single dose	3	39 (14.0)	3 (100%)	3 (100%)	0 (0%)	0 (0%)	23.4 (3.01)	–	–	–	–
	Inclisiran 800 single dose	6	49 (6.7)	5 (83.3%)	3 (50.0%)	0 (0%)	1 (16.7%)	25.9 (1.60)	–	–	–	–
	Placebo with statin (multiple dose)	4	58 (3.0)	2 (50.0%)	4 (100%)	0 (0%)	0 (0%)	26.5 (2.72)	–	–	–	–
	Placebo without statin (multiple dose)	8	51 (14.2)	6 (75.0%)	7 (87.5%)	0 (0%)	0 (0%)	26.7 (2.64)	–	–	–	–
	Inclisiran 300 mg with Statin	4	52 (21.6)	2 (50.0%)	3 (75.0%)	0 (0%)	1 (25.0%)	27.1 (3.59)	–	–	–	–
	Inclisiran 300 mg without statin	6	47 (8.7)	6 (100%)	6 (100%)	0 (0%)	0 (0%)	25.2 (2.95)	–	–	–	–
	Inclisiran 500 mg with statin	5	56 (11.5)	2 (40.0%)	3 (60.0%)	1 (20.0%)	1 (20.0%)	25.7 (1.97)	–	–	–	–
	Inclisiran 500 mg without statin	6	42 (16.1)	3 (50.0%)	5 (83.3%)	0 (0%)	1 (16.7%)	23.0 (2.34)	–	–	–	–
	Inclisiran 125 mg without Statin	6	55 (9.4)	4 (66.7%)	5 (83.3%)	0 (0%)	1 (16.7%)	26.2 (2.72)	–	–	–	–
	Inclisiran 250 without statin	6	61 (6.3)	4 (66.7%)	3 (50.0%)	1 (16.7%)	0 (0%)	27.0 (1.93)	–	–	–	–
Ray et al. [26]	Placebo single dose	105	62.7 (10.6)	45(45.9%)	94 (95.9%)	–	–	31.1 (5.2)	66 (67.3%)	67 (68.4%)	15 (19.4%)	17 (17.3%)
	300 mg of inclisiran single dose	203	63.6 (9.2)	50 (47.6%)	101 (96.2%)	–	–	32.2 (6.8)	66 (62.9%)	79 (75.2%)	16 (15.2%)	13 (12.4%)
Ray et al. [24]	ORION 10 trial		–		–	–	–	–	–	–	–	–
	placebo	780	66.4(8.9)	535(68.5%)	653 (83.6%)	–	–	–	371 (47.5%)	714 (91.4%)	123 (15.7%)	8 (1.0%)
	300 mg of inclisiran single dose	781	65.7(8.9)	548(70.3%)	685 (87.8%)	–	–	–	331 (42.4%)	701 (89.9%)	111 (14.2%)	12 (1.5%)
	ORION 11 TRIAL		–	–	–	–	–	–	–	–	–	–
	placebo	807	64.8(8.3)	579(71.5%)	791 (97.7%)	–	–	–	296 (36.5%)	640 (79.0%)	160 (19.8%)	14 (1.7%)
	300 mg of inclisiran single dose	810	64.8(8.7)	581(72%)	796 (98.6%)	–	–	–	272 (33.7%)	601 (81.9%)	132 (16.4%)	14 (1.7%)
Raal et al. [25, 41]	placebo	240	55.5(4.6)	112(46.3%)	226 (93.4%)	–	–	–	20 (8.3%)	102 (42.1%)	28 (11.6%)	–
	300 mg inclisiran sodium	242	55.(5.1)	115(47.9%)	227 (94.6%)	–	–	–	28 (11.7%)	101 (42.1%)	28 (11.7%)	–
Nissen et al. [23]	placebo	8	52.9(12)	2 (25%)	6 (75%)	1(13%)	1(13%) (Asian)	25(4)	0 (0%)	1(13%)	–	–
	30 mg SLN360	6	45(10.5)	4 (67%)	1 (17%)	5 (83%)	0 (0%)	26(3.3)	0 (0%)	0 (0%)	–	–
	100 mg SLN360	6	46.3(12.3)	4 (67%)	5 (83%)	1 (17%)	0 (0%)	–	–	–	–	–
	300 mg SLN360	6	58.7(13.2)	2 (33%)	3 (50)	3(50%)	0(0%)	29(4)	2(33%)	0 (0%)	–	–
	600 mg SLN360	6	43.7(17.5)	3 (50%)	5 (83%)	1(13%)	0 (0%)	27(3.8)	1(17%)	0 (0%)	–	–
O’Donoghue et al. [22]	Placebo	54	63.4(8.9)	36 (67%)	48(89%)	2(4%)	1(2%)	–	38(70%)	12(22%)	–	9(17%)
	Olpasiran 10 mg, Every 12 Weeks	58	63.4(9.5)	46 (79%)	52(90%)	0 (0%)	0(0%)	–	37(64%)	8(14%)	–	9(16%)
	Olpasiran 75mg, Every 12 Weeks	58	61.3(9.2)	35 (60%)	52 (90%)	1 (2%)	0(0%)	–	38(66%)	14(24%)	–	11(19%)
	Olpasiran 225 mg, Every 12 Weeks	56	59.7(10.1)	41 (73%)	47 (84%)	2 (4%)	2(4%)	–	39(70%)	9(16%)	–	5(9%)
	Olpasiran 225 mg, Every 24 Weeks	55	61.8(9.4)	33 (60%)	49 (89%)	1 (2%)	0(0%)	–	32(58%)	7(13%)	–	15(27%)
Sohn et al. [28]	Total (Japanese + non-Japanese)	27	48(12.5)	17 (63%)	4(14.8%)	1(16.7%)	1(16.7%)	23.9(3.2)	–	–	–	–
	Japanese Olpasiran 3 mg	6	56(4.6)	2 (33.3%)	0 (0%)	0 (0%)	0(%)	22.7(3.7)	–	–	–	–
	Japanese Olpasiran 9mg	6	49(9.7)	3 (50%)	0 (0%)	0 (0%)	0(0%)	24.1(2.7)	–	–	–	–
	Japanese Olpasiran 75 mg	5	45.2(13.7)	5 (100%)	0 (0%)	0 (0%)	5(0%)	23.7(3.4)	–	–	–	–
	Japanese Olpasiran 225 mg	4	56.3(4.9)	1 (25%)	0 (0%)	0 (0%)	0(0%)	23.4(3.7)	–	–	–	–
	Total for Japanese	21	51.5(9.6)	11 (52.4%)	0 (0%)	0 (0%)	0()%)	23.5(3.1)	–	–	–	–
	Non-Japanese Olpasiran 75 mg	6	36(14.8)	6 (100%)	4(66.7%)	1 (3.7%)	1(3.7%)	25.6(3)	–	–	–	–
Koenig et al. [27]	placebo Polyvascular disease	242	66.9(8.7)	168(69.4%)	–	–	–	30.4(5.6)	223(92.1%)	106(43.8%)	52(21.5%)	–
	300 mg inclisiran sodium Polyvascular disease	228	67.4(8.5)	152(66.7%)	–	–	–	30.3(6.1)	206(90.4%)	118(51.8%)	51(22.4%)	–
	placebo non-Polyvascular disease	1478	63.4(10)	1025(69.4%)	–	–	–	30.7(5.8)	1159(78.4%)	459(31.1%)	203(13.7%)	–
	300 mg inclisiran sodium non-Polyvascular disease	1506	63.7(10.1)	1029(68.3%)	–	–	–	30.4(5.6)	1182(78.5%)	503(33.4%)	241(16%)	–
Nissen et al. [18, 42]	Placebo	12	50.3(11.1)	6(50%)	6(50%)	4(33.3%)	–	29.4(5.8)	–	–	–	–
	Lepodisiran 4mg	6	40.5(11.7)	5(83.3%)	0 (0%)	4(66.7%)	–	29.8(6.7)	–	–	–	–
	Lepodisiran 12mg	6	44.3(8.3)	5(83.3%)	0 (0%)	4(66.7%)	–	26(6.9)	–	–	–	–
	Lepodisiran 32mg	6	50.7(11.3)	3(50%)	3(50%)	2(33.3%)	–	29(6.7)	–	–	–	–
	Lepodisiran 96mg	6	47.8(10.4)	3(50%)	3(50%)	2(33.3%)	–	27.4(4.8)	–	–	–	–
	Lepodisiran 304mg	6	51.8(9.5)	4(66.7%)	2(33.3%)	3(50.7%)	–	25.2(3.7)	–	–	–	–
	Lepodisiran 608mg	6	38.5(15.6)	5(83.3%)	2(33.3%)	4(66.7%)	–	27.7(5)	–	–	–	–

**Fig. 3:**
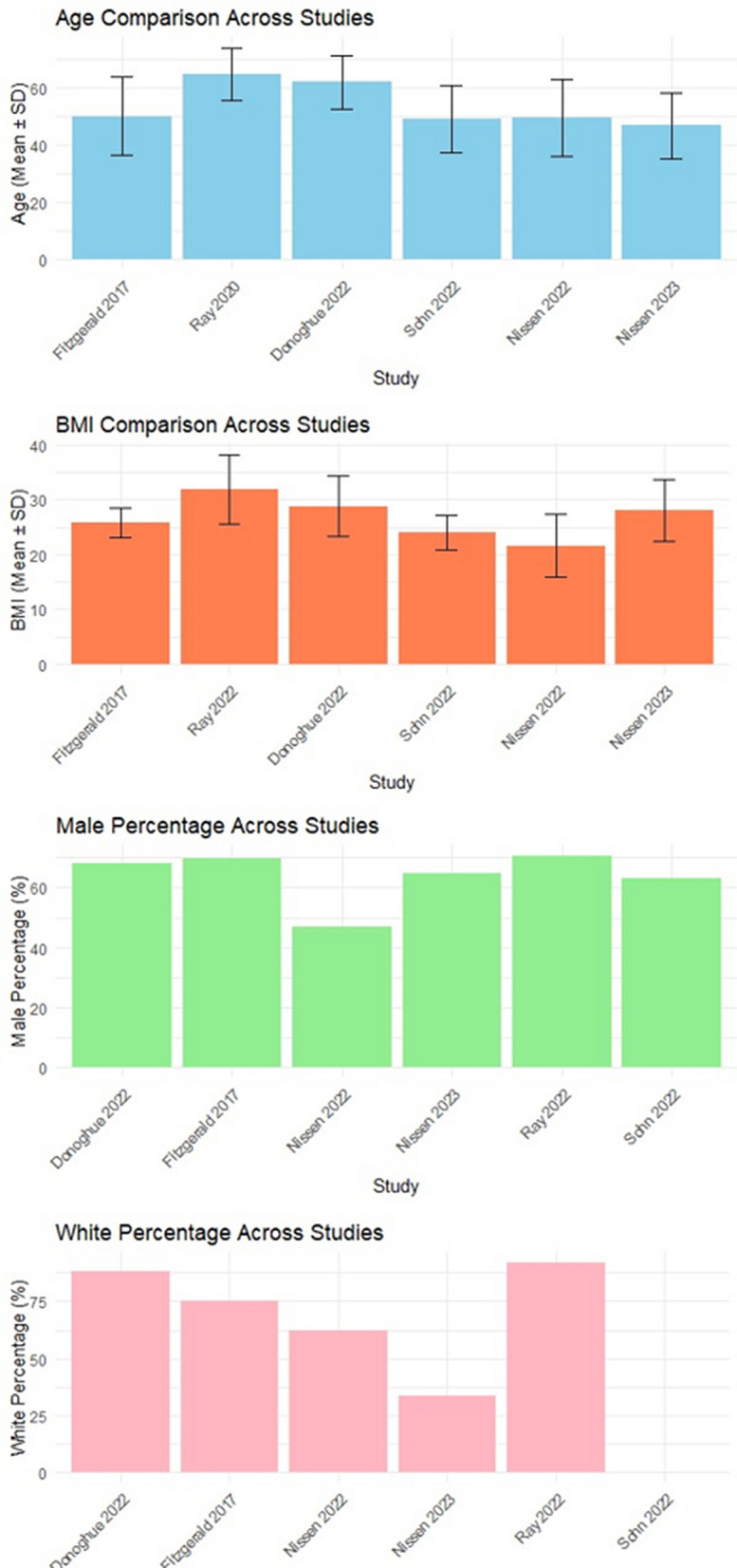
Baseline characteristics (age, BMI, gender and race) across studies

The studies were performed with different baseline body mass index (BMI), including > 25 kg/m^2^ [18, 23, 26, 27, 29] and < 25 kg/m^2^ [13]. Regarding race, the participants in some studies were only white [24–26], and in other studies, they included different races, including white, black, and others, such as Hispanics [18, 22, 23, 28, 29] (Table 1 and Fig. 3). All included studies provided information on the dosage used in their interventions. There were no uniform doses used across the different RCTs. The inclisiran dose varied between 25 and 800 mg [24, 26, 27, 29] while olpasiran doses ranged from 3 to 225 mg [22, 28], and SLN360 ranged from 30 to 600 mg [23], and only one trial assessed lepodisiran with the following doses 4 mg, 12 mg, 32 mg, 96 mg, 304 mg, and 608 mg [18].

The lipid measures identified at baseline in these studies were the total cholesterol level [18, 23–27], Lp (a) [18, 22–28], LDL-C [18, 22–25, 27, 29], triglycerides [18, 23–26, 29], HDL-C [18, 23–25, 27], Apo-B gl-1 [18, 22–27], and PCSK9 antibodies [22, 24–27, 29], while the lipid therapies used by the participants were statins [22–27, 29] and Ezetimibe [22, 24–27] (Supplementary Table 1). Four studies included LDL-C levels at least 100 mg/dl or higher [25–27, 29], while one study included patients whose LDL-C was > 70 mg/dl [24]. Two studies included LP(a) over 150 nmol/l [22, 23]. One study included patients with Lp(a) > 75 nmol/l [18]. No entry threshold criterion regarding Lp(a) level was applied in one study [28]. 

### Safety outcomes

Most of the studies included reported adverse events ranging from minor to serious adverse events (Supplementary Table 2). Overall, injection site reactions were the most common adverse event reported in the trials, with 7 out of 8 studies reporting this event; these reactions were more common in treatment groups compared to placebo groups, which occurred in about 5% of patients in treatment groups; however, these reactions were mainly mild and transient, and none of them were severe or persistent [22–28].

Five studies illustrated the adverse events of inclisiran versus placebo in patients with atherosclerotic cardiovascular disease or with high risk, most adverse events in each study were reported to be mild to moderate, with no treatment discontinuation that occurred due to adverse events [24–27, 29]. Two inclisiran studies included severe and fatal adverse events such as liver injury (defined as an increase in AST or ALT three above the limit of normal or bilirubin two above the limit of normal) and kidney adverse events (defined as increasing creatinine > 2 mg/dl), thrombocytopenia and hyperglycemia [24, 26]. The olpasiran studies showed low-risk adverse events with no significant safety concerns [22, 28].

Nissen et al. focused on treatment-emergent adverse events in SLN360 groups which were generally mild; one participant had values for AST and ALT that were greater than three times the upper limit of normal [23].

Regarding lepodisiran, Injection site reactions, mainly pain, were common in all groups (different doses) but mostly resolved before discharge [18]. Moreover, two participants experienced elevated aminotransaminase levels; three patients had high creatine kinase levels, in which all were transient, with no systemic hypersensitivity or cytokine-release syndrome observed [18].

### Efficacy outcomes

All studies focused on determining the efficacy of SIRNA therapies on LP(a) and other lipids reduction such as LDL-C, APO B, triglycerides, HDL-C, total cholesterol, and non-HDL-C (Supplementary Table 3).

#### Reduction in LP(a) level

Five studies focused on inclisiran, and three of these studies pointed out the effectiveness of inclisiran on LP(a) levels reduction; Ray et al. highlighted that the mean percent change in LP(a) concentration from day 90 to 540 showed a decreased of − 12.1 (95% CI [− 20.8 to − 3.4]), in contrast to increase in the Lp(a) in the placebo group (*P* value < 0.0001) [26]. This observation underscores the effectiveness of inclisiran in reducing lipid concentrations compared to placebo [26], while Koenig et al. [27] and Fitzgerald et al. [29] provided insights into the impact of inclisiran on LP(a) as a secondary outcome. The remaining two studies did not report any reduction in LP(a) levels [24, 25].

Two studies focused on olpasiran showed a reduction in Lp(a) [22, 28]. Sohn et al. reported that Lp(a) levels were reduced in a dose-dependent manner, as from day 29 to 255 in the olpasiran 3 mg group with the mean percent change in Lp(a) concentration was − 19.5 (ranging from − 23.6 to − 14), while in olpasiran 225 mg it was − 89.1 (ranging from − 95 to − 82.96) [28], while In Dongue et al., in the olpasiran10 mg Every 12 week yielded mean change in Lp(a) concentration of − 66.9 (95% CI [− 70.4 to − 63.4]) and the 225 mg every 24 week yielded − 96.9 (95% CI [− 100.0 to − 93.3]) [22]. These studies also demonstrated a reduction of Lp(a) in a dose-dependent manner [22, 28].

#### Reduction in LP(a) level between the Japanese and non-Japanese groups

Sohn et al. [28] found that after a single dose of 75 mg olpasiran, the magnitude and durability of Lp(a) were similar between the Japanese and non-Japanese groups with the same mean baseline Lp(a) level (33 nmol/L) and the same mean percentage reduction from baseline in Lp(a) at day 57 (95%) [28].

Nissen et al. [23] focused on SLN 360, demonstrating that the absolute and percentage changes in Lp(a) were dose-dependent. This study also noted that although LP(a) concentrations gradually increased from their lowest point (nadir) after medication usage, however, they did not return to baseline levels.

Most recently, a new medication called lepodisiran deemed to be very effective in lowering Lp(a), with the median percentage changes for each treatment group from baseline to day 337 was as follows: − 5% (IQR − 16 to 11%) in the placebo group − 41% (IQR − 47% to − 20%) in the 4 mg of lepodisiran group, − 59% (IQR − 66% to − 53%) in the 12-mg dose group, − 76% (IQR − 76% to − 75%) in the 32-mg dose group, − 90% (IQR − 94% to − 85%) in the 96-mg dose group, − 96% (IQR − 98% to − 95%) in the 304-mg dose group, and − 97% (IQR − 98% to − 96%) in the 608-mg dose group[18].

**Fig. 4:**
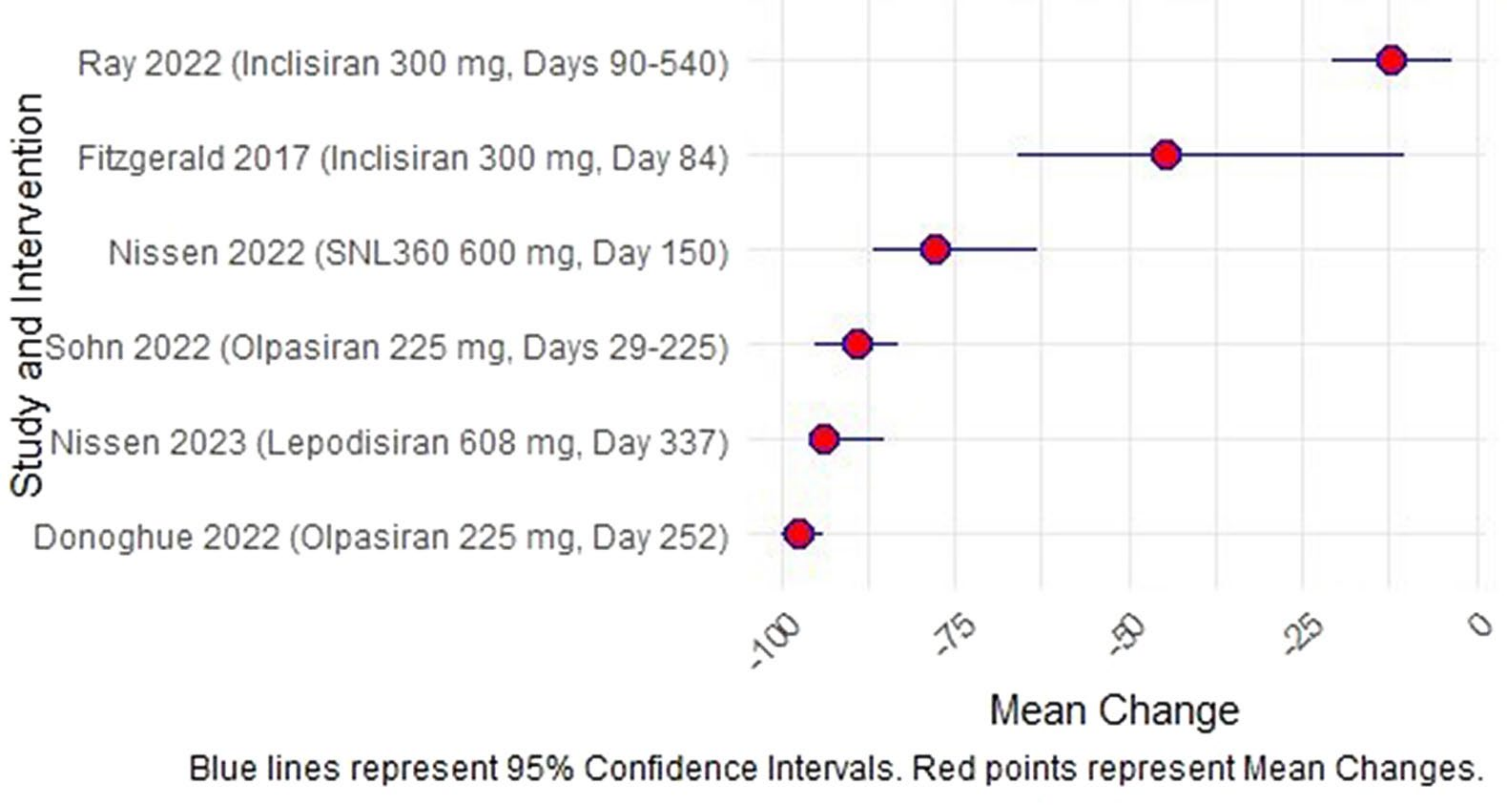
Mean Lp(a) reduction across studies

Overall, the reduction in Lp(a) differs significantly between studies with certain doses of olpasiran and lepodisiran showing the most reduction (Fig. 4).

#### Reduction in LDL-C level

Five studies focused on inclisiran showed a significant reduction in LDL-C levels [24–27, 29]. The average reduction shown was 36.7–59.7%, they also demonstrated that LDL-C reduction is higher in inclisiran groups than in the placebo [24–27, 29]. Two studies illustrated the effect of olpasiran on LDL-C levels [22, 28], O'Donoghue et al. showed a reduction in the placebo-adjusted mean percent change of LDL-C; the average reduction was 22.6–24.8% [28] while Sohn et al. showed no reduction in LDL-C levels [22]. In Nissen et al., SLN 360 produced a dose-dependent reduction in LDL-C, and the mean levels were reduced by 26% [23]. Inclisiran is more effective in lowering LDL-C levels compared to olpasiran and SLN360.

#### Reduction in APO B level

Ray et al. [26], Koing et al. [27] and Fitzgerald et al. [29] showed that patients treated with inclisiran had a decrease in ApoB levels. O’Donoghue et al. reported that the placebo-adjusted mean percent change in the ApoB concentration ranged from − 16.7 to − 18.9% across olpasiran different doses [22], while Nissen et al. focused on SLN360, with the maximum reduction in the mean level of apolipoprotein was 24% [23].

#### Reduction in other lipids

Ray et al. [26] showed that patients treated with inclisiran had a reduction in non-HDL-C and total cholesterol levels, and it showed an increase in HDL-C levels [26]. Fitzgerald et al. showed a decrease in total cholesterol and non-HDL-C [29]. Six studies did not show any change in HDL-C, non-HDL-C, total cholesterol, or triglyceride.

## Discussion

Lp(a) has gained much attention recently as it is considered an independent risk factor for CAD; when elevated, it is considered a risk factor even in patients receiving cholesterol-lowering therapy [5]. Moreover, around 20% of the population will have high Lp(a) > 150 nmol/l or > 50 mg/dl [43, 44]. Moreover, patients with familial hypercholesterolemia (FH) are specifically at risk as a mild increase of Lp(a) as low as 30 mg/dl increases the risk of CAD [5]. Hence, there is a pressing need for medications that lower Lp(a) and more research to assess its effect on mortality and morbidity.

With modern healthcare focusing on chronic disease prevention, adding new medications to our arsenal of lipid-lowering therapies is an important topic. In this systematic review, we focused on studies that explored the potential use of siRNA on LDL-C and Lp(a) reduction.

Most of the included RCTs in this systematic review demonstrated the efficacy of siRNA in reducing Lp(a). Studies showed that inclisiran, one of the well-studied SiRNA, demonstrated dose-dependent reduction going up to 300 mg single dose, with a mean percentage change from baseline of 44.5% in a healthy population, however, increasing the dose above 500 failed to demonstrate any efficacy in lowering Lp(a) [29]. In another study, a dose of 300 mg in adults with patients with ASCVD showed only a 12.1% reduction from baseline [26]; this indicates that more studies investigating the optimal dose of inclisiran are needed, especially in a different population with various risk factors.

While olpasiran and SLN360 successfully demonstrated dose-dependent effects in healthy adults and adults with ASCVD [22, 23, 28], interestingly, olpasiran reduced the Lp(a) more effectively than inclisiran and SLN360. Hence, if the goal is to reduce Lp(a), olpasiran may be a better choice. However, further comparative studies and phase 3 trials are needed to confirm such findings and to compare safety profiles in well-randomized individuals/populations.

While the effect on LDL reduction was more robust, inclisiran showed a dose-dependent reduction in LDL-C, with the 500 mg dosage achieving the most significant mean percent decrease from baseline at 50.6%. At the same time, a further increase in the dose did not result in LDL-c reduction [29]. In the ORION-10 and ORION-11 trials, inclisiran yielded a mean LDL cholesterol level reduction from the baseline of 51.3% and 45.8%, respectively, substantially more significant than the 1% and 4% reductions in their respective placebo groups [24]. Furthermore, in patients with familial hypercholesterolemia, inclisiran 300 mg showed 39.7% LDL-c reduction [25]. The inclisiran effect is not only limited to healthy individuals but extends to individuals with polyvascular disease (e.g., peripheral artery disease, coronary artery disease, or cerebrovascular disease) [27]. Inclisiran was more effective in reducing LDL-C than olpasiran and SLN360. Hence, it may be used if further LDL-c reduction is needed.

SiRNA medications are broadly considered safe, with most adverse events being mild to moderate, rarely leading to treatment discontinuation. However, serious complications, including liver and kidney issues, thrombocytopenia, and hyperglycemia, were reported in some inclisiran studies [24–27, 29]. Olpasiran studies showed mostly low-risk adverse events, with musculoskeletal pain and mild liver enzyme elevation among the noted effects [22, 28]. However, overall adverse event rates were comparable to the placebo group’s [22, 28]. Injection site reactions emerged as the most common side effect, affecting about 5% of participants, but were generally mild and short-lived [22, 28]. Regarding SLN360 therapy, it also showed that 75–100% of participants experienced mild to moderate adverse events, including low-grade injection site reactions, headache, and diarrhea [23]. Lepodisiran, a new SiRNA therapy studied in individuals from the USA and Singapore, was deemed to be generally safe for individuals with Lp(a) > 75 nmol/l, with only one unrelated adverse severe event and minor, self-resolving case of elevated aminotransferases and creatine kinase [18]. The study highlighted injection site pain/erythema as a common side effect [18]. Overall, most siRNA medications are deemed to be generally safe, but monitoring for side effects is crucial. Moreover, the long-term side effects are yet to be discovered, and studies with long-term follow-up are required.

Given the genetics role in the elevated Lp(a), it is worth mentioning that individuals with FH in whom a little rise in the Lp(a), as much as 30 mg/dl, put them at higher risk of CAD [5, 8]. FH is a severe autosomal dominant genetic disorder characterized by elevated LDL-C levels, predisposing individuals to premature CAD [45]. FH is caused by mutations in LDL receptors, ApoB, or PCSK9 genes, with patients classified as having heterozygous (HeFH) or homozygous (HoFH) forms depending on whether one or both alleles are affected [46, 47]. Current treatments for FH aim to lower LDL-C levels through lifestyle modifications, lipoprotein apheresis, and pharmacological interventions, including statins, PCSK9 inhibitors, and ezetimibe [48]. The reduction in LDL cholesterol levels of almost 50% with twice-yearly administration of inclisiran in patients with heterozygous FH who had been receiving maximally accepted background statin therapy has the potential to improve their adherence to the treatment regimen [41]. Such advancements in hyperlipidemia treatment could bridge critical gaps in managing both LDL-C and Lp(a)-driven cardiovascular risks, especially in high-risk populations like those with FH.

This comprehensive review underscores the promising value of siRNA therapies in reducing Lp(a) and other lipids for dyslipidemia treatment while highlighting the necessity for further research to address safety concerns and efficacy. It is a critical reference for future clinical trials, aiming to refine our understanding of siRNA therapy's role in managing cardiovascular diseases.

However, challenges still exist as different populations have different Lp(a) thresholds contributing to cardiovascular disease [5], However, the included studies show heterogeneity in different aspects such as drug dosage, follow-up duration, baseline Lp(a) levels, and patient population. The variability in drug dosing complicates the direct comparison of efficacy across studies. Drug dosage varied, with inclisiran ranging from 25 to 800 mg, while SLN360 and olpasiran were also tested at different doses. Additionally, follow-up duration ranged from 150 to 540 days. These differences in follow-up time frames could influence the long-term assessment of drug efficacy.

Regarding baseline lipoprotein(a) levels, studies like Nissen et al. [23] and O’Donoghue et al. [22] included patients with high Lp(a) levels (> 125 nmol/l) [49], while Ray et al. [24, 26], Raal et al. [25, 41], Sohn et al. [28], and Koenig et al. [27] included patients with lower Lp(a) levels (< 125 nmol/l).

In terms of the included population, O’Donoghue et al. [22] and Ray et al. [24, 26] investigated patients with diabetes or atherosclerotic cardiovascular disease (ASCVD), whereas Fitzgerald et al. [29] and Raal et al. [25, 41] concentrated on patients with LDL < 100 mg/dl. Koenig et al. [27] investigated people with polyvascular disease (PVD), while Nissen et al. [23] and Sohn et al. [28] focused on individuals without cardiovascular disease. These data in detail are provided in supplementary Tables 1 and 5. Additionally, all the clinical trials were conducted in a few countries, which may limit the generalizability of their findings to other populations. However, this was improved; for example, the phase 1 RCT that studied lepodisiran was more inclusive of Asian, Black, and white populations. The heterogeneity in dosages, follow-up, Lp(a), and patient populations makes the direct comparison between the studies more challenging, so this is an essential factor for future clinical trials.

Further work is also needed to define the role of Lp(a) reduction in patients with and without cardiovascular disease to determine if the drug can prevent the development of atherosclerotic disease, in which long-term follow-up studies will be beneficial. Eventually, the studies included were mainly phase 1 and 2 RCTs. Given the small sample size and a limited number of studies that focused primarily on Lp(a) reduction, there is a pressing need for high-quality RCT studies with larger sample sizes to evaluate the efficacy and safety of siRNA therapies on Lp(a) reduction.

Future studies should focus on assessing the long-term safety profile of siRNA particularly the chronic side effects, organ-specific toxicities, and potential immune response that may emerge after chronic use. Additionally, long-term efficacy should be evaluated to ascertain whether treatment advantages are sustainable and to keep an eye out for any delayed negative effects, especially in populations with underlying health conditions. Future research should also investigate the ideal dosage, patent adherence, and long-term drug interaction. Such investigations are important to ensure the safety and efficacy of siRNA in clinical practice.

Eleven ongoing trials are exploring various siRNA medications and their role in lipid reduction, and their results will be very enlightening. These ongoing studies encompass various international clinical trials (Australia, USA, China, Hong Kong, Brazil) focusing on cardiovascular health. They primarily investigate the efficacy of drugs like SLN360, olpasiran, and inclisiran in affecting lipid profiles, particularly lipoprotein(a) and LDL-C, in individuals with various cardiovascular risk factors (Supplementary Table 5). 

## Conclusion

This systematic review highlights the significant potential of small interfering RNA (siRNA) therapies, such as inclisiran, SLN360, and olpasiran, in the management of dyslipidemia, specifically targeting lipoprotein(a) (Lp(a)) and LDL-C reduction. The Lp(a) reduction was dose-dependent, with Olpasiran showing the most promising results. Moreover, siRNA has generally favorable safety profiles, with injection site reaction being the most reported adverse event. Despite the promising results, the review identifies the critical need for more high-quality large scales studies to advance our knowledge of the pathophysiological role of defined Lp(a) in atherosclerotic cardiovascular disease and clarify the effectiveness of siRNA therapies. In addition, these studies should include diverse populations to confirm these findings and ascertain siRNA therapies' long-term adverse events. Such future research is imperative to fully establish the role of siRNA therapies in cardiovascular disease management and its contribution to the prevention of chronic cardiovascular conditions, thereby enhancing global cardiovascular health outcomes. 

## Supplementary Information

Additional file 1.

## Data Availability

Not applicable.

## Abbreviations

ASO: Antisense oligonucleotide
ASCVD: Atherosclerotic cardiovascular disease
CAD: Coronary artery disease
HDL-C: High-density lipoprotein cholesterol
HMG-CoA: 3-Hydroxy-3-methyl-glutaryl-coenzyme A
Lp(a): Lipoprotein(a)
LDL-C: Low-density lipoprotein cholesterol
MeSH: Medical subject headings
mRNA: Messenger RNA
PRISMA: Preferred reporting items for systematic review
PCSK-9: Proprotein convertase subtilisin/kexin type 9
RCTs: Randomized controlled trials
siRNA: Small interfering RNA
