# Supplementary material for: Small interfering RNA effect on lipoprotein(a): a systematic review
Source: Egypt Heart J. 2025 May 8;77:44. doi: 10.1186/s43044-025-00635-1 (PMC12062474; doi:10.1186/s43044-025-00635-1)
Supplement: Supplementary file 1 — Additional file 1. [file 43044_2025_635_MOESM1_ESM.docx]

|  |
| --- |

Supplementary

Supplementary table 1: Lipid measures and lipid therapy.

Supplementary table 2: Safety outcomes of the included studies.

Supplementary table 3: Efficacy outcomes of the included studies.

Supplementary table 4: Details of the included randomized controlled trials (RCTS).

Supplementary table 5: Characteristics of the included ongoing studies.

Supplementary table 6: Cochrane Risk of Bias Tool for Randomized Controlled Trials (ROB)

Supplementary table 7: Detailed search strategy

**Supplementary table 1: Lipid measures and lipid therapy.**

| PCSK9 monoclonal antibody | | Lipid measures (mean/SD) | | | | | | | | | | | | Lipid therapy (N%) | | | | | | Intervention name | | Study ID  (author/year) | |
| --- | --- | --- | --- | --- | --- | --- | --- | --- | --- | --- | --- | --- | --- | --- | --- | --- | --- | --- | --- | --- | --- | --- | --- |
|  | | APO B gl-1 | | HDL-c  (mg/dl) | | Triglycerides  (mg/dl) | | LDL-c  (mg/dl) | | Lipoprotein (a)  (nmol/l) | | Total cholesterol  (mg/dl) | | Lipid modifying therapy | | | | Lipid lowering therapy | |  | |  | |
|  |  |  |  |  |  |  |  |  |  |  |  |  |  | Ezetimibe | | statins | |  |  |  |  |  |  |
| 278.95 (99.53) | | --- | | --- | | 70.9 (12.4) | | 131.5 (19.3) | | --- | | --- | | --- | | 0 (0%) | | 0(0%) | | Placebo single dose | | Fitzgerald et.al, 2017 | |
| 342.65 (67.89) | | --- | | --- | | 115.1 (59.3) | | 177.9 (50.7) | | --- | | --- | | --- | | 0 (0%) | | 0(0%) | | Inclisiran 25 mg single | |  |  |
| 233.77 (39.17) | | --- | | --- | | 177.1 (102.7) | | 150.8 (35.6) | | --- | | --- | | --- | | 0 (0%) | | 0(0%) | | Inclisiran 100 single dose | |  |  |
| 253.82 (22.36) | | --- | | --- | | 132.9 (48.7) | | 162.4 (36.7) | | --- | | --- | | --- | | 0 (0%) | | 0(0%) | | Inclisiran 300 single dose | |  |  |
| 263.23 (24.98) | | --- | | --- | | 159.4 (84.1) | | 119.9 (17.0) | | --- | | --- | | --- | | 0 (0%) | | 0(0%) | | Inclisiran 500 single dose | |  |  |
| 279.62 (66.90) | | --- | | --- | | 115.1 (21.3) | | 158.5 (28.6) | | --- | | --- | | --- | | 0 (0%) | | 0(0%) | | Inclisiran 800 single dose | |  |  |
| 460.69 (56.295) | | --- | | --- | | 150.6 (46.9) | | 143.1 (89.7) | | --- | | --- | | --- | | 4 (100%) | | 4 (100%) | | Placebo with statin (multiple dose) | |  |  |
| 276.23 (58.69) | | --- | | --- | | 124.0 (38.1) | | 131.5 (20.9) | | --- | | --- | | --- | | 0 (0%) | | 0(0%) | | Placebo without statin (multiple dose) | |  |  |
| 460.69 (209.435) | | --- | | --- | | 132.9 (86.8) | | 143.1 (30.5) | | --- | | --- | | --- | | 4 (100%) | | 4 (100%) | | Inclisiran 300 mg with statin | |  |  |
| 311.47 (59.85) | | --- | | --- | | 132.9 (90.3) | | 143.1 (20.1) | | --- | | --- | | --- | | 0 (0%) | | 0(0%) | | Inclisiran 300 mg without statin | |  |  |
| 433.44 (107.28) | | --- | | --- | | 97.4 (44.3) | | 104.4 (19.7) | | --- | | --- | | --- | | 5 (100%) | | 5 (100%) | | Inclisiran 500 mg with statin | |  |  |
| 288.07 (69.07) | | --- | | --- | | 88.6 (20.4) | | 123.7 (49.9) | | --- | | --- | | --- | | 0 (0%) | | 0(0%) | | Inclisiran 500 mg without statin | |  |  |
| 380.03 (50.63) | | --- | | --- | | 88.6 (25.7) | | 139.2 (18.6) | | --- | | --- | | --- | | 0 (0%) | | 0(0%) | | Inclisiran 125 mg with statin | |  |  |
| 288.73 (53.53) | | --- | | --- | | 159.4 (69.1) | | 146.9 (14.3) | | --- | | --- | | --- | | 0 (0%) | | 0(0%) | | Inclisiran 250 with statin | |  |  |
| --- | | --- | | --- | | --- | | --- | | --- | | --- | | --- | | --- | | --- | | ORION 10 trial | | Ray et.al,2020 | |
| 422.1±176.9 | | 94.1±25.6 | | 46.6±14.3 | | 113.2±47.0 | | 104.5±39.6 | | 78.2±47.0 | | 180.6±46.1 | | 80(10.2%) | | 701(89.8%) | | --- | | 300 mg of inclisiran single dose | |  |  |
| 414.9±145.7 | | 94.6±25.1 | | 45.9±14.4 | | 116.7±48.8 | | 104.8±37 | | 80.2±48.8 | | 180.6±43.6 | | 74(9.5%) | | 692(88.7%) | | --- | | placebo | |  |  |
| --- | | --- | | --- | | --- | | --- | | --- | | --- | | --- | | --- | | --- | | ORION 11 trial | |  |  |
| 355±98.9 | | 97.1±28.0 | | 49.7±15.8 | | 116.5±46.2 | | 107.2±41.8 | | 70.0±46.2 | | 187.3±48.2 | | 52(6.3%) | | 766(94.6%) | | --- | | 300 mg of inclisiran single dose | |  |  |
| 353±97.4 | | 95.1±5.2 | | 45.3±13.8 | | 117.3±47.0 | | 103.7±36.4 | | 67.2±47.0 | | 183.3±42.8 | | 62(77%) | | 766(94.9%) | | --- | | placebo | |  |  |
| 452.2±131.2 | | 123.8±33.2 | | 51.5±15.1 | | 122.2±24.5 | | 151.4±50.4 | | 79±45.6 | | 230±54.6 | | 135(55.8%) | | 219(90.5%) | | --- | | 300 mg Inclisiran sodium | | Raal et.al,2022 | |
| 429.1±135.3 | | 124.5±34.8 | | 50.8±13.1 | | 122.2±23.4 | | 154.7±58.0 | | 78.2±47.6 | | 232.4±62.8 | | 120(50.0%) | | 217(90.4%) | | --- | | placebo | |  |  |
| --- | | 83(23) | | 70(14) | | Median (IQR) 55(34-71) | | 113(38) | | Median (IQR)  171(142-219) | | 197(47) | | --- | | 0(0%) | | --- | | 30 mg SLN36 | | Nissen et.al, 2022 | |
| --- | | 94(29) | | 55(14) | | 80(63-130) | | 121(46) | | 217(202-274) | | 195(47) | | --- | | 2(33%) | | --- | | 100 mg SLN360 | |  |  |
| --- | | 89(6) | | 46(9) | | 85(67-179) | | 100(25) | | 285(195-338) | | 170(19) | | --- | | 3(50%) | | --- | | 300 mg SLN360 | |  |  |
| --- | | 81(25) | | 55(9) | | 75(42-115) | | 108(54) | | 231(179-276) | | 180(51) | | --- | | 3(50%) | | --- | | 600 mg SLN360 | |  |  |
| --- | | 81(30) | | 67(20) | | 93(59-122) | | 99(48) | | 238(203-308) | | 185(54) | | --- | | 5(63%) | | --- | | Placebo | |  |  |
| 12(22) | | Median (IQR) 62.5(48.5–76.0) | | --- | | --- | | Median (IQR) 64.8(47.5–81.0) | | Median (IQR) 246.1(199.9–343.3) | | --- | | 22(41%) | | 45(83%) | | --- | | placebo | | Donoghue et.al,2022 | |
| 15(26) | | 66.8(51.5–81.5) | | --- | | --- | | 69(52.0–83.5) | | 304(194.2–397.6) | | --- | | 32(55%) | | 52(90%) | | --- | | Olpasiran 10 mg, Every 12 Weeks | |  |  |
| 11(19) | | 74(59.5–85.0 | | --- | | --- | | 75(53.5–90.0) | | 227.5(188.4–304.2) | | --- | | 31(53%) | | 50(86%) | | --- | | Olpasiran 75mg, Every 12 Weeks | |  |  |
| 16(29) | | 62.8(49.5-80.8) | | --- | | --- | | 62.3(48.5-80.5) | | 265.4(200.6-342.2) | | --- | | 26(46%) | | 51(91%) | | --- | | Olpasiran 225 mg, Every 12 Weeks | |  |  |
| 12(22) | | 64(56.5–79.0) | | --- | | --- | | 66(50.5–79.5) | | 283.4(204.6–389.2) | | --- | | 36(65%) | | 49(89%) | | --- | | Olpasiran 225 mg Every 24 Weeks | |  |  |
| --- | | --- | | --- | | --- | | --- | | 41.1(34.4) | | --- | | --- | | --- | | --- | | Japanese Olpasiran 3 mg | | Sohn et.al, 2022 | |
| --- | | --- | | --- | | --- | | --- | | 96.9(71) | | --- | | --- | | --- | | --- | | Japanese Olpasiran 9 mg | |  |  |
| --- | | --- | | --- | | --- | | --- | | 32.9(45.2) | | --- | | --- | | --- | | --- | | Japanese Olpasiran 75 mg | |  |  |
| --- | | --- | | --- | | --- | | --- | | 34.7(18.8) | | --- | | --- | | --- | | --- | | Japanese Olpasiran 225 mg | |  |  |
| --- | | --- | | --- | | --- | | --- | | 53.9(52.9) | | --- | | --- | | --- | | --- | | Total for Japanese | |  |  |
| --- | | --- | | --- | | --- | | --- | | 33(39.9) | | --- | | --- | | --- | | --- | | Non-Japanese Olpasiran 75 mg | |  |  |
| --- | | --- | | --- | | --- | | --- | | 49.2(50.4) | | --- | | --- | | --- | | --- | | Total | |  |  |
| 404.3(111.3) | | 93.4(28.3) | | 45.7(13.9) | | 151.1(77.6) | | 102.2(44.8) | | Median (IQR) 93.0 (24-200) | | 178.0(50.2) | | 22 (9.6%) | | 211 (92.5%) | | --- | | 300 mg Inclisiran sodium Polyvascular disease | | Koeing et.al, 2022 | |
| 394.5(174.6) | | 95.2(26.6) | | 47.4(15.4) | | 149.4(79.8) | | 104.0(38.1) | | 51.5 (19-194) | | 181.6(44.9) | | 33 (13.6%) | | 222 (91.7%) | | --- | | placebo Polyvascular disease | |  |  |
| 397.4(152.9) | | 98.7(27.9) | | 48.9(15.0) | | 145.6(74.8) | | 111.2(42.0) | | 45.0 (18-183 | | 189.1(47.7) | | 225 (14.9%) | | 1395 (92.6%) | | --- | | 300 mg Inclisiran sodium non Polyvascular disease | |  |  |
| 390.8(122.1) | | 98.0±28.1 | | 47.9±13.8 | | 147.2(77.1) | | 110.1(43.5) | | 47.0 (19-185) | | 187.3(48.5) | | 229 (15.5%) | | 1362 (92.2%) | | --- | | placebo non Polyvascular disease | |  |  |
| 122.2±40.5 | | 1.3±0.4 | | --- | | 3.7±1.7 | | --- | | 41.1(34.4) | | 6.0±1.8 | | 4(4.1%) | | 89(84.8%) | | --- | | 300 mg Inclisiran sodium | | Ray et.al,2022 | |
| 117.4±29.8 | | 1.3±0.4 | | --- | | 3.5±1.2 | | --- | | 96.9(71) | | 5.8±1.5 | | 7(6.7%) | | 79(80.6%) | | --- | | placebo | |  |  |
| --- | | 123(30) | | 49(10) | | 116(88-193) | | 143(36) | | 111(78-134) | | 219(46) | | --- | | --- | | --- | | placebo | | Nissen et.al,2023 | |
| --- | | 90(22) | | 58(19) | | 76(58-89) | | 108(34) | | 78(50-152) | | 182(32) | | --- | | --- | | --- | | Lepodisiran 4mg | |  |  |
| --- | | 117(38) | | 54(15) | | 105(57-167) | | 148(38) | | 97(86-107) | | 224(43) | | --- | | --- | | --- | | Lepodisiran 12mg | |  |  |
| --- | | 93(13) | | 48(9) | | 70(56-109) | | 110(13) | | 120(110-188) | | 176(16) | | --- | | --- | | --- | | Lepodisiran 32mg | |  |  |
| --- | | 97(17) | | 58(12) | | 75(72-81) | | 118(17) | | 167(124-189) | | 191(14) | | --- | | --- | | --- | | Lepodisiran 96mg | |  |  |
| --- | | 114(22) | | 50(17) | | 92(81-125) | | 142(27) | | 96(72-132) | | 214(43) | | --- | | --- | | --- | | Lepodisiran 304mg | |  |  |
| --- | | 108(32) | | 52(20) | | 103(86-119) | | 135(31) | | 130(87-181) | | 212(50) | | --- | | --- | | --- | | Lepodisiran 608mg | |  |  |

**Supplementary table 2: Safety outcomes of the included studies.**

| Study (author/year) | Intervention name | Treatment-emergent adverse events | | | Injection site reaction N (%) | Myalgia N (%) | Liver-related adverse event N (%) | Kidney-related adverse event N (%) | Thrombocytopenia N (%) | Hyperglycemia N (%) |
| --- | --- | --- | --- | --- | --- | --- | --- | --- | --- | --- |
|  |  | Any N (%) | Serious N (%) | Fatal N (%) |  |  |  |  |  |  |
| Fitzgerald et.al, 2017 | Placebo single dose | 2 (33.3%) | --- | --- | --- | 1 (33.3%) | --- | --- | --- | --- |
|  | Inclisiran 25 mg single | 2 (66.7%) | --- | --- | --- | 0(0%) | --- | --- | --- | --- |
|  | Inclisiran 100 single dose | 1 (33.3%) | --- | --- | --- | 0(0%) | --- | --- | --- | --- |
|  | Inclisiran 300 single dose | 1 (33.3%) | --- | --- | --- | 0(0%) | --- | --- | --- | --- |
|  | Inclisiran 500 single dose | 1 (33.3%) | --- | --- | --- | 0(0%) | --- | --- | --- | --- |
|  | Inclisiran 800 single dose | 4 (66.7%) | --- | --- | --- | 1 (16.7%) | --- | --- | --- | --- |
|  | Placebo with statin (multiple dose) | 4 (100%) | --- | --- | --- | --- | --- | --- | --- | --- |
|  | Placebo without statin (multiple dose) | 5 (62.5%) | --- | --- | --- | --- | --- | --- | --- | --- |
|  | Inclisiran 300 mg with statin | 3 (75.0%) | --- | --- | --- | --- | --- | --- | --- | --- |
|  | Inclisiran 300 mg without statin | 2 (33.3%) | --- | --- | --- | --- | --- | --- | --- | --- |
|  | Inclisiran 500 mg with statin | 5 (100%) | --- | --- | --- | --- | --- | --- | --- | --- |
|  | Inclisiran 500 mg without statin | 4 (66.7%) | --- | --- | --- | --- | --- | --- | --- | --- |
|  | Inclisiran 125 mg with statin | 5 (83.3%) | --- | --- | --- | --- | --- | --- | --- | --- |
|  | Inclisiran 250 with statin | 5 (83.3%) | --- | --- | --- | --- | --- | --- | --- | --- |

| Ray et.al,2022 | Placebo single dose | 91(92.9%) | 20(20.4%) | 1(1.0%) | 4(4.1%) | CK >5 UNL 3(3.1%) | ALT >3ULN 1(1.0%),  AST >3ULN 0,  Bilirubin >2 ULN 1(1.0%) | creatinine >2mg/dl =0 | 0(0%) | --- |
| --- | --- | --- | --- | --- | --- | --- | --- | --- | --- | --- |
|  | 300 mg of inclisiran single dose | 88(83.8%) | 13(12.4%) | 1(1.0%) | 0(0%) | CK >5 UNL 1(1.0%) | ALT >3ULN 1(1.0%),  AST >3ULN 1(1.0%),  Bilirubin >2 ULN 1(1.0%) | creatinine >2mg/dl =2(1.9%) | 0(0%) | --- |
| Ray et.al,2020 | ORION 10 trial | | | | | | | | | |
|  | placebo | 582(74.8%) | 205(26.3%) | 11(1.4%) | 7(0.9%) | ck>5ULN 8(1.0%) | ALT >3 ULN 2(0.3%),  AST >3 ULN 5(0.6%),  Bilirubin >2 ULN 3(0.4%) | creatinine >2mg/dl =3(0.4%) | 0(0%) | 108(13.9%) |
|  | 300 mg of inclisiran single dose | 574(73.5%) | 175(22.4%) | 12(1.5%) | 20(2.6%) | ck>5ULN 10(1.3%) | ALT >3 ULN 2(0.3%),  AST >3 ULN 4(0.5%),  Bilirubin >2 ULN 4(0.5%) | creatinine >2mg/dl =4(0.5%) | 1(0.1%) | 120(15.4%) |
|  | ORION 11 TRIAL | | | | | | | | | |
|  | placebo | 655(81.5%) | 181(22.5%) | 15(1.9) | 4(0.5) | ck>5ULN 9(1.1%) | ALT >3 ULN 4(0.5%),  AST >3 ULN 4(0.2%),  Bilirubin >2 ULN 8(1.0%) | creatinine >2mg/dl =8(1.0%) | 1(0.1%) | 94(11.7) |
|  | 300 mg of inclisiran single dose | 671(8.7%) | 181(22.3%) | 14(1.7) | 38(4.7) | ck>5ULN 10(1.2%) | ALT >3 ULN 4(0.5%),  AST >3 ULN 2(0.2%),  Bilirubin >2 ULN 6(0.7%) | creatinine >2mg/dl =6(0.7%) | 0(0) | 88(10.9) |
| Raal et.al,2020 | placebo | 172(71.7%) | 33(13.8%) | 1(0.4%) | 4(1.7%) | ck.5UNL 5(2.1%) | ALT >3 ULN 2(0.8%),  AST >3 ULN 1(0.4%),  Bilirubin>2 ULN 3(1.2%) | creatinine >2ULN 1(0.4%) | 1(0.4%) | --- |
|  | 300 mg inclisiran sodium | 185(76.8%) | 18(7.2%) | 1(0.4%) | 41(17%) | ck.5UNL 4(1.7%) | ALT >3 ULN 3(1.2%),  AST >3 ULN 1(0.4%),  Bilirubin >2 ULN 4(1.7%) | creatinine >2ULN 1(0.4%) | 0(0%) | --- |
| Nissen et.al, 2022 | placebo | 6(75%) | 0(0%) | NA | 1(13%) | NA | 0 (0%) | --- | --- | --- |
|  | 30mg SLN360 | 6(100%) | 1(17%) | NA | 5(83%) | NA | ALT >3× ULN= 1 (17%) AST >3× ULN= 1 (17%) | --- | --- | --- |
|  | 100 mg SLN360 | 6(100%) | 0(0%) | NA | 6(100%) | NA | 0(0%) | --- | --- | --- |
|  | 300 mg SLN360 | 6(100%) | 0(0%) | NA | 5(83%) | NA | 0(0%) | --- | --- | --- |
|  | 600 mg SLN360 | 6(100%) | 0(0%) | NA | 6(100%) | NA | 0(0%) | --- | --- | --- |
| Donoghue et.al,2022 | placebo | 45(83%) | 8(15%) | 1(2%) | 6(11%) | 4(7%) | 2(4%) | 1(2%) | 1(2%) | 3(6%) |
|  | Olpasiran 10 mg, Every 12 Weeks | 45(78%) | 3(5%) | 0(0%) | 3(5%) | 3(5%) | 1(2%) | 0(0%) | 0(0%) | 5(9%) |
|  | Olpasiran 75mg, Every 12 Weeks | 46(79%) | 3(5%) | 0(0%) | 11(19%) | 1(2%) | 2(3%) | 1(2%) | 0(0%) | 3(5%) |
|  | Olpasiran 225 mg, Every 12 Weeks | 47(84%) | 6(11%) | 0(0%) | 12(21%) | 4(7%) | 1(2%) | 0(0%) | 0(0%) | 5(9%) |
|  | Olpasiran 225 mg Every 24 Weeks | 47(85%) | 4(7%) | 0(0%) | 13(24%) | 4(7%) | 1(2%) | 0(0%) | 0(0%) | 3(5%) |
|  | Total | 185(81%) | 16(7%) | 0(0%) | 39(17%) | 12(5%) | 5(2%) | 1 (<1%) | 0(0%) | 16(7%) |

| Sohn et.al, 2022 | Japanese Olpasiran 3 mg | 2(33.3%) | 0(0%) | 0(0%) | --- | 1(16.7) | --- | --- | --- | --- |
| --- | --- | --- | --- | --- | --- | --- | --- | --- | --- | --- |
|  | Japanese Olpasiran 9 mg | 4(66.7%) | 0(0%) | 0(0%) | --- | 0(0) | --- | --- | --- | --- |
|  | Japanese Olpasiran 75 mg | 0(0%) | 0(0%) | 0(0%) | --- | 0(0) | --- | --- | --- | --- |
|  | Japanese Olpasiran 225 mg | 3(75%) | 0(0%) | 0(0%) | --- | 0(0) | --- | --- | --- | --- |
|  | Non-Japanese Olpasiran 75 mg | 0(0%) | 0(0%) | 0(0%) | --- | 0(0) | --- | --- | --- | --- |
| Koeing et.al, 2022 | 300 mg Inclisiran sodium Polyvascular disease | 186 (81.2%) | 70 (30.6%) | --- | 7 (3.1%) | --- | 1 (0.4%) | 17 (7.4%) | 0 (0.0%) | --- |
|  | placebo Polyvascular disease | 202 (83.8%) | 93 (38.6%) | --- | 0 (0.0%) | --- | 1 (0.4%) | 13 (5.4%) | 1 (0.4%) | --- |
|  | 300 mg Inclisiran sodium non Polyvascular disease | 1152 (76.5%) | 283 (18.8%) | --- | 80 (5.3%) | --- | 7 (0.5%) | 30 (2.0%) | 1 (0.1%) | --- |
|  | placebo non Polyvascular disease | 1117 (75.8%) | 313 (21.2%) | --- | 12 (0.8%) | --- | 5 (0.3%) | 32 (2.2%) | 1 (0.1%) | --- |
| Nissen et.al,2023 | placebo | --- | .0(0%) | --- | 2(33.3%) | CK=0 | ALT >3× ULN=0  AST >3× ULN=0  Bilirubin= 0 | --- | --- | --- |
|  | Lepodisiran 4mg | --- | 1(16.6%) | --- | 0 (0%) | CK=2 | ALT >3× ULN=0  AST >3× ULN=1  Bilirubin= 0 | --- | --- | --- |
|  | Lepodisiran 12mg | --- | 0(0%) | --- | 3 (50%) | CK=0 | ALT >3× ULN=0  AST >3× ULN=0  Bilirubin= 0 | --- | --- | --- |
|  | Lepodisiran 32mg | --- | 0(0%) | --- | 2 (33.3%) | CK=0 | ALT >3× ULN=0  AST >3× ULN=0  Bilirubin= 0 | --- | --- | --- |
|  | Lepodisiran 96mg | --- | 0(0%) | --- | 2 (50%) | CK=0 | ALT >3× ULN=1  AST >3× ULN=1  Bilirubin= 0 | --- | --- | --- |
|  | Lepodisiran 304mg | --- | 0(0%) | --- | 3 (50%) | CK=0 | ALT >3× ULN=0  AST >3× ULN=0  Bilirubin= 0 | --- | --- | --- |
|  | Lepodisiran 608mg | --- | 0(0%) | --- | 3(50%) | CK=1 | ALT >3× ULN=0  AST >3× ULN=0  Bilirubin= 0 | --- | --- | --- |

**Supplementary table 3: Efficacy outcomes of the included studies.**

| Study  (author/ year) | Intervention name | The maximum absolute change in Lp(a) | maximum mean percent change from baseline of lp(a) | Lp(a) mean percent change from baseline | Mean percent change from baseline in corrected LDL-c | Mean percent change from baseline in Apo B | Mean percent change from baseline in HDL-C | Mean percent change from baseline in triglycerides | Mean percent change of total cholesterol | Mean percent change of NON-HDL-C |
| --- | --- | --- | --- | --- | --- | --- | --- | --- | --- | --- |
|  |  |  |  |  |  |  |  |  |  |  |
| Fitzgerald et.al,2017 | Placebo single dose | --- | --- | Day 84    3.6 (−28.6 to 50.2) | Day 84 -10.9(-26.0 to 7.1) | Day 84 −15.3 (−31.4 to 4.6) | Day 84 13.6 (−9.2 to 42.1) | --- | Day 84 −4.4 (−15.9 to 8.7) | Day 84 −11.7 (−23.7 to 2.3) |
|  | Inclisiran 25 mg single | --- | --- | Day 84 −14.0 (−55.1 to 64.6) | Day 84 -21.5(-41.3 to 5.0) | Day 84 −11.3 (−36.8 to 24.5) | Day 84 7.3 (−22.0 to 47.7) | --- | Day 84 −12.0 (−30.1 to 10.8) | Day 84 −19.8 (−36.1 to 0.6) |
|  | Inclisiran 100 single dose | --- | --- | Day 84 −20.8 (−51.0 to 28.0) | Day 84 -36.7(-50.2 to -19.4) | Day 84 −26.5 (−43.3 to −4.7) | Day 84 17.9 (−9.2 to 53.1) | --- | Day 84 −17.7 (−30.0 to −3.4) | Day 84 −28.4 (−40.8 to −13.5) |
|  | Inclisiran 300 single dose | --- | --- | Day 84 −44.5 (−65.7 to −10.4) | Day 84 -50.0(-60.7 to -36.3) | Day 84 −47.1 (−59.0 to −31.9) | Day 84 36.8 (2.9 to 82.0) | --- | Day 84 −30.9 (−41.2 to −18.9) | Day 84 −48.9 (−57.8 to −38.2) |
|  | Inclisiran 500 single dose | --- | --- | Day 84 −35.5 (−60.1 to 4.3) | Day 84 -50.6(-61.3 to -36.9) | Day 84 −39.9 (−53.9 to −21.7) | Day 84 7.4 (−17.4 to 39.8) | --- | Day 84 −27.1 (−38.2 to −14.0) | Day 84 −36.3 (−47.4 to −22.9) |
|  | Inclisiran 800 single dose | --- | --- | Day 84 −21.3 (−45.0 to 12.6) | Day 84 -43.4(-52.5 to -32.4) | Day 84 −37.5 (−47.7 to −25.2) | Day 84 0.1 (−16.8 to 20.5) | --- | Day 84 −29.1 (−36.8 to −20.6) | Day 84 −37.0 (−44.9 to −28.0) |
|  | Placebo with statin (multiple dose) | --- | --- | Day 84 | Day 84 -14.2(-30.2 to 5.5) | Day 84 −12.8 (−23.2 to −1.0) | Day 84 0.6 (−6.0 to 7.6) | --- | Day 84 −7.6 (−15.5 to 1.0) | Day 84 −10.6 (−21.3 to 1.6) |
|  | Placebo without statin (multiple dose) | --- | --- | --- | --- | --- | --- | --- | --- | --- |
|  | Inclisiran 300 mg with statin | --- | --- | Day 84 −6.0 (−26.7 to 20.4) | Day 84 -45.1(-61.6 to -21.4) | Day 84 −37.2 (−49.8 to −21.4) | Day 84 10.8 (−1.5 to 24.6) | --- | Day 84 -24.9 (−35.8 to −12.0) | Day 84 −35.7 (−48.7 to −19.3) |
|  | Inclisiran 300 mg without statin | --- | --- | Day 84 −30.7 (−54.3 to 5.1) | Day 84 -59.7(-68.7 to- 48.1) | Day 84 −52.4 (−59.4 to −44.2) | Day 84 11.7 (2.7 to 21.4) | --- | Day 84 −40.4 (−46.7 to −33.4) | Day 84 −56.9 (−63.3 to −49.4) |
|  | Inclisiran 500 mg with statin | --- | --- | Day 84 −19.2 (−40.0 to 8.8) | Day 84 -53.2(-64.5 to -38.3) | Day 84 −41.8 (−51.1 to −30.7) | Day 84 5.3 (−3.9 to 15.3) | --- | Day 84 −30.4 (−38.4 to −21.3) | Day 84 −46.2 (−54.9 to −35.8) |
|  | Inclisiran 500 mg without statin | --- | --- | Day 84 −42.7 (−59.7 to −18.4) | Day 84 -51.7(-62.5 to -37.8) | Day 84 −46.4 (−54.3 to −37.2) | Day 84 12.8 (3.8 to 22.6) | --- | Day 84 −27.0 (−34.7 to −18.3) | Day 84 −45.1 (−53.3 to −35.5) |
|  | Inclisiran 125 mg with statin | --- | --- | Day 84 −27.4 (−45.8 to −2.7) | Day 84 -39.8(-51.1 to -25.9) | Day 84 −33.3 (−43.1 to −21.8) | Day 84 12.9 (3.8 to 22.7) | --- | Day 84 −23.8 (−31.9 to −14.7) | Day 84 −36.9 (−46.3 to −25.9) |
|  | Inclisiran 250 with statin | --- | --- | Day 84 −22.7 (−28.4 to −16.5) | Day 84 -52.2(-62.9 to -38.4) | Day 84 −46.5 (−54.3 to −37.3) | Day 84 4.1 (−4.2 to 13.1) | --- | Day 84 −34.5 (−41.4 to −26.7) | Day 84 −45.3 (−53.4 to −35.8) |
| Ray et.al 2022 | Placebo single dose | Day (90 to 540)  5.5 (1.2to 9.9) | --- | Day (90 to 540)  16.8 (8.5, 25.1) | Day (90 to 540)  0.6 (−4.1to 5.3) | Day (90 to 540)  −0.7 (−4.5, 3.0) | Day (90 to 540)  6.0 (3.1to 8.9) | Day (90 to 540)  2.5 (−3.2 to 8.2) | Day (90 to 540)  0.3 (−3.0 to3.6) | Day (90 to 540)  −0.2 (−4.5 to 4.2) |
|  | 300mg of inclisiran single dose | Day (90 to 540)  −12.5 (−17.1to −8.0) | --- | Day (90 to 540)  −12.1 (−20.8 to −3.4) | Day (90 to 540)  −40.4 (−45.3, −35.5) | Day (90 to 540)  −35.5 (−39.5, −31.6) | Day (90 to 540)  11.0 (8.0, 14.1) | Day (90 to 540)  −5.9 (−11.9 to 0.0) | Day (90 to 540)  −24.7 (−28.2 to −21.3) | Day (90 to 540)  −35.5 (−40.0 to −30.9) |

| Ray et.al,2020 | ORION 10 trial | --- | --- | Day (baseline to 510) | Day (baseline to 510) | --- | --- | --- | --- | --- |
| --- | --- | --- | --- | --- | --- | --- | --- | --- | --- | --- |
|  | placebo | --- | --- | +3.7% | 1.2(-1.6 to 4.09) | --- | --- | --- | --- | --- |
|  | 300 mg of inclisiran single dose | --- | --- | -21.9% | -51.3(-55.7 to -48.8) | --- | --- | --- | --- | --- |
|  | ORION 11 TRIAL | --- | --- | Day (baseline to 510) | Day (baseline to 510) | --- | --- | --- | --- | --- |
|  | placebo | --- | --- | 0% | 4(1.8 to 6.1) | --- | --- | --- | --- | --- |
|  | 300 mg of inclisiran single dose | --- | --- | -18.6% | -45.8(-53.1 to -46.6) | --- | --- | --- | --- | --- |
| Raal et.al,2020 | placebo | --- | --- | Day (baseline to 510)  +3.7% | Day (baseline to 510)  8.2(4.3 to 12.2) | --- | --- | --- | --- | --- |
|  | 300 mg of Inclisiran sodium | --- | --- | -13.5% | Day (baseline to 510)  -39.7(-43.7 to 35.7) | --- | --- | --- | --- | --- |
| Nissen et.al, 2022 | placebo | Day 150 -20(-61 to3) | Day 150 -10(-16 to 1) | Day 150 -4.08(-14.5 to 7.9) | Day 150 -12.19(-4.7 to 30) | Day 150 -6.9(-26.7 to 13.1) | --- | --- | --- | --- |
|  | SLN 360 30mg | Day150 -89(-119 to -61) | Day 150 -46(-64 to -40) | Day 150 -5.2(-16.12 to -1.6) | Day 150 8.7(-7.8 to 25.4) | Day 150 6.2(-1.1 to 13.8) | --- | --- | --- | --- |
|  | SLN360 100mg | Day 150 -185(-226 to -163) | Day 150 -86(-92 to -82) | Day 150 -34.9(-50.2 to -28.1) | Day 150 -1.9(-25.4 to 20.6) | Day 150 6.9(-4.5 to 18.7) | --- | --- | --- | --- |
|  | SLN360 300mg | Day 150 -268(-292 to -189) | Day 150 -96(-98 to -89) | Day 150 -66.7(-82.7 to -45.4) | Day 150 -1.09(-24.5 to 24.6) | Day 150 -1.09(-13.8 to 11.8) | --- | --- | --- | --- |
|  | SLN360 600mg | Day 150 -227(-270 to -174) | Day 150 -98(-98 to -97) | Day 150 -77.9(-86.7 to -63.07) | Day 150 -10.9(41.5 to 20.3) | Day 150 -5.4(-23.6 to 13.1) | --- | --- | --- | --- |
| Donoghue et.al,2022 | placebo | --- | --- | Week 36 3.6 (−0.1 to 7.3) | Week 36 6.3 (−2.6 to 15.2) | Week 36 7.4 (1.4 to 13.4) | --- | --- | --- | --- |
|  | Olpasiran 10 mg, Every 12 Weeks | --- | --- | Week 36 −66.9 (−70.4 to −63.4) | Week 36 −17.4 (−25.8 to −9.1) | Week 36 −11.5 (−17.2 to −5.8) | --- | --- | --- | --- |
|  | Olpasiran 75mg, Every 12 Weeks | --- | --- | Week 36 −93.8 (−97.3 to −90.3) | Week 36 −16.3 (−24.6 to −7.9) | Week 36 −9.3 (−15.0 to −3.6) | --- | --- | --- | --- |
|  | Olpasiran 225 mg, Every 12 Weeks | --- | --- | Week 36 −97.5 (−100.0 to −94.0) | Week 36 −16.8 (−25.4 to −8.1) | Week 36 −10.2 (−16.0 to −4.4) | --- | --- | --- | --- |
|  | Olpasiran 225 mg Every 24 Weeks | --- | --- | Week 36 −96.9 (−100.0 to −93.3) | Week 36 −18.5 (−27.1 to −9.8) | Week 36 −11.4 (−17.3 to −5.5) | --- | --- | --- | --- |
|  | total | --- | --- | --- | --- | --- | --- | --- | --- | --- |
| Sohn et.al, 2022 | Japanese Olpasiran 3 mg | --- | At day 57 -55(-64 to -47.2) | Day (29 to 225)  -19.5(-23.6 to -14) | --- | --- | --- | --- | --- | --- |
|  | Japanese Olpasiran 9 mg | --- | At day 57 -79.6(-86 to -72.3) | Day (29 to 225)  -38.8(-54 to -23.6) | --- | --- | --- | --- | --- | --- |
|  | Japanese Olpasiran 75 mg | --- | At day 57 -94(-96 to -92) | Day (29 to 225)  -67.4(-71.9 to -62.6) | --- | --- | --- | --- | --- | --- |
|  | Japanese Olpasiran 225 mg | --- | At day 57 -98(-99.2 to -98.2) | Day (29 to 225)  -89.1(-95 to -82.96) | --- | --- | --- | --- | --- | --- |
|  | Non-Japanese Olpasiran 75 mg | --- | At day 57 -94(-96 to -92) | Day (29 to 225)  -73.6(-78.1 to -68.5) | --- | --- | --- | --- | --- | --- |

| Study  (author /year) | Intervention  name | The maximum absolute change in Lp(a) | Maximum mean percent change from baseline of Lp(a) | Lp(a) mean percent change from baseline | Mean percent change from baseline in corrected LDL-C | Mean percent change from baseline in APO-B | Mean percent change from baseline in HDL-C | Mean percent change from baseline in triglyceride | Mean percent change from baseline in total cholesterol | Mean percent change from baseline in non-HDL-C |
| --- | --- | --- | --- | --- | --- | --- | --- | --- | --- | --- |
| Koeing et .al 2022 | placebo Polyvascular disease | --- | --- | Day 510 4.8 (0.2, 9.5) | Day 510 1.2 (−5.2, 7.6) | Day 510 3.0 (−1.6, 7.5) | --- | Day 510 −2.7 (−4.6, −10.0) | Day 510 3.8 (−0.1, 7.7) | Day 510 5.6 (0.3, 10.8) |
|  | 300 mg Inclisiran sodium Polyvascular disease | --- | --- | Day 510 −16.8 (−21.9, −11.6) | Day 510 −15.3 (−21.9, −8.6) | Day 510 −39.7 (−44.4, −34.9) | --- | Day 510 −14.6 (−22.2, −7.0) | Day 510 −28.8 (−32.9, −24.7) | Day 510 −42.3 (−47.7, −36.8) |
|  | placebo non Polyvascular disease | --- | --- | Day 510 4.3 (2.7, 5.9) | Day 510 2.6 (0.7, 4.6) | Day 510 1.7 (0.5, 2.8) | --- | Day 510 3.3 (1.1, 5.6) | Day 510 2.9 (1.9, 4.0) | Day 510 3.5 (2.1, 4.9) |
|  | 300 mg Inclisiran sodium non Polyvascular disease | --- | --- | Day 510 −16.1 (−17.8, −14.3) | Day 510 −6.3 (−8.2, −4.4) | Day 510 −40.5 (−41.6, −39.3) | --- | Day 510 −5.3 (−7.5, −3.0) | Day 510 −29.8 (−30.8, −28.7) | Day 510 −43.1 (−44.6, −41.7) |
| Nissen et.al2023 | placebo | Maximum median change  Day 337 -3(-18 to 12) | Maximum median percent  Day 337 -5(-16 to 11) | --- | --- | --- | --- | --- | --- | --- |
|  | Lepodisiran 4mg | Day 337 -36(-46 to -12) | Day 337 -41(-47 to -20) | --- | --- | --- | --- | --- | --- | --- |
|  | Lepodisiran 12mg | Day 337 -58(-63 to -45) | Day 337 -59(-66 to -53) | --- | --- | --- | --- | --- | --- | --- |
|  | Lepodisiran 32mg | Day 337 -85(-86 to -84) | Day 337 -76(-76 to -75) | Day 337 -27(-41 to -16) | --- | --- | --- | --- | --- | --- |
|  | Lepodisiran 96mg | Day 337 -155(-160 to -117) | Day 337 -90(-94 to -85) | Day 337 -58(-71 to -51) | --- | --- | --- | --- | --- | --- |
|  | Lepodisiran 304mg | Day 337 -111(-126 to -74) | Day 337 -96(-98 to -95) | Day 337 -75(-85 to -69) | --- | --- | --- | --- | --- | --- |
|  | Lepodisiran 608mg | Day 337 -127(-147 to -84) | Day 337 -97(-98 to -96) | Day 337 -94(-94 to -85) | --- | --- | --- | --- | --- | --- |

**Supplementary table 4: Details of the included randomized controlled trials (RCTS).**

| Study  (author/year) | Study design | Sample size | Duration | Patient or disease | Drug used | Study arms | Inclusion criteria | Exclusion criteria | Primary outcome | Secondary  outcome | conclusion |
| --- | --- | --- | --- | --- | --- | --- | --- | --- | --- | --- | --- |
| Fitzgerald et.al, 2017 | Randomized, single-blind, placebo-controlled study | 69 | 210 days | Patients with LDL-C higher than 100 mg/dl or triglycerides less than 400 mg/dl. | Inclisiran or placebo | inclisiran or placebo in either a single-dose phase (at a dose of 25, 100, 300, 500, or  800mg) or a multiple-dose phase (125 mg, 250 mg  , or 300 or 500 mg, with or without concurrent statin therapy) | Patients with LDL-C higher than 100 mg/dl or triglycerides less than 400 mg/dl. | --- | -LDL-C  -PCSK9 | -LDL-C  -PCSK9  -total cholesterol  -non-HDL-C  -apolipoprotein(B)  -HDL-C  -lipoprotein (a) | Inclisiran reduced levels of pcsk9 and LDL-C for at least 6 months, but it didn’t cause any serious events. |
| Ray et.al,2022 | a randomized, double-blind, placebo-controlled, Phase 3 trial | 203 | 540 days | Patients with atherosclerotic cardiovascular diseases or its equivalent as DM. | inclisiran sodium | subcutaneous inclisiran sodium 300 mg vs. placebo | patients with LDL-C higher than 70 mg/dl in presence of cardiovascular diseases and higher than 100 mg/dl in absence of it. | Any use of a monoclonal antibody drug targeting PCSK9 at any time | -LDL-C  -Lipoprotein(a)  -Non HDL-C  -HDL-C  -Total cholesterol  -Apolipoprotein (B) | -LDL-C  -Lipoprotein(a)  -Non HDL-C  -HDL-C  -Total cholestérol  -Apolipoprotein B | Inclisiran was good for primary prevention patients with elevated LDL-C and reduce the atherogenic lipoproteins with 2 doses per year. |
| Ray et.al,2020 | two randomized, double-blind, placebo-controlled, parallel-group, phase 3 trials. | 3178 | 540 days | Patients with atherosclerotic cardiovascular disease | Inclisiran or placebo | inclisiran (284 mg) or matching placebo | patients with LDL-C higher than 70 mg/dl in presence of (CVS) diseases and higher than 100 mg/dl in absence of it. They get the maximum accepted dose of a statin with or without lipid-lowering therapy for at least 30 days before screening, with no plan to change therapy during trial. | Any use of a monoclonal antibody drug targeting PCSK9 within 90 days before screening. | -LDL-C | -LDL-C  -PCSK9  -Total cholesterol  -Non-HDL  -Lipoprotein(a)  -apolipoprotein(B)  -HDL-C  -triglycerides | There was a reduction in LDL-C level with the administration of Inclisiran for 6 months of about 50%. It also caused injection site adverse effect more than placebo. |
| Raal et.al,2020 | double-blind, randomized, placebo-controlled trial | 482 | 540 days | Patients with LDL-C 100mg/dl or more despite receiving statin therapy with or without ezetimibe | Inclisiran sodium or placebo | inclisiran sodium (at a dose of 300 mg,)  or matching placebo | Patients with LDL-C 100mg/dl or more despite receiving statin therapy with or without ezetimibe | Any use of a monoclonal antibody drug targeting PCSK9 | -LDL-C  -PCSK9 | -LDL-C  -lipoprotein(a)  -Apolipoprotein (B)  -Non-HDL-C  -HDL-C  -Triglyceride  -Total cholesterol | Adults with familial hypercholesterolemia  Who received inclisiran show less levels of LDL-C with infrequent doses and accepted safety profile. |
| Nissen et.al, 2022 | Randomized controlled trial | 32 | 150 days | adults with no cardiovascular disease | SLN 360 | 30-mg SLN360  100-mgSLN360  300-mg SLN360  600-mg SLN360  281Or placebo | Adults with no cardiovascular disease, with BMI 18 to 45 and LP(a) 150mmol/l or more | any patient with moderate to severe hepatic sclerosis, positive HCV, HBV or HIV. Other liver disease or drugs induce liver injury. | Adverse events of each treatment dose | -Lipoprotein(a)  -Total cholesterol  -LDL-C  -Exploratory outcomes (apolipoprotein B) | The SiRNA SLN360 was well tolerated and used in lowering of plasma Lp(a) concentrations. |
| Donoghue et.al,2022 | multicenter, randomized, double-blind, placebo-controlled, dose-finding trial | 281 | 48 weeks | Adults with history of cardiovascular disease. | Olpasiran | Olpasiran 10 mg,  Every 12 weeks  Olpasiran 75 mg,  Every 12 weeks  Olpasiran 225 mg Every 12 weeks  Olpasiran 225 mg,  Every 24 weeks  Placebo | Adults with history of cardiovascular disease and concentration of LP(a) more than 150mmol/l | severe renal dysfunction or a history or clinical evidence of active liver disease. | -Lipoprotein (A) | -Lipoprotein (A)  LDL-C  -Aolipoprotein (B) | Olpasiran therapy reduced lipoprotein(a) concentrations in patients with atherosclerotic cardiovascular disease. Other trials will be done to determine its effect on cardiovascular disease. |
| Sohn et.al, 2022 | Randomized control trial (phase one open label parallel group single dose study) | 75 | April 2019 to May 2020 | Adults aged 18 to 60 years with BMI between 18 and 32 | Olpasiran | Japanese Olpasiran 3 mg  Japanese Olpasiran 9 mg - Japanese Olpasiran 75 mg Japanese Olpasiran 225 mg  Non-Japanese Olpasiran 75 mg | Adults aged 18 to 60 years with BMI between 18 and 32.  Japanese participants were born in Japan and were first generation Japanese. | History of peripheral neuropathy or malignancy, bleeding or coagulation disorder, diabetes mellitus, the use of apheresis as lipid lowering therapy 14 days before the study. | -PK parameters | Exploratory endpoints  -Lipoprotein(a)  -Total cholesterol  -LDL-C  -HDL-C  -Triglycerides  -Apolipoprotein (B)  Apolipoprotein(a) | Olpasiran was well tolerated without serious adverse events or laboratory or vital signs abnormalities. |
| Koeing et.al, 2022 | randomized, double-blinded, placebo-controlled trials – post-hoc analysis of ORION 9, 10 and 11. | 3454 | 540 days | Patients with polyvascular disease (PVD) or at risk | inclisiran | Inclisiran PVD  Placebo PVD  Inclisiran non-PVD  Placebo non-PVD | Participants older than 18 years old with history of heart failure, atherosclerotic cardiovascular disease or its equivalent | Any use of a monoclonal antibody drug targeting PCSK9 within 90 days of screening. | -LDL-C | -LDL-C  -PCSK9  -Total cholesterol  -Lipoprotein(a)  -Apolipoprotein (B)  -Non HDL-C | Two doses of inclisiran per year were well tolerated and help effectively to lower lipids in patients regardless the PVD status. |
| Nissen et.al,2023 | A single ascending-dose trial | 48 | 337 days | 48 adults without cardiovascular disease aged of 18 and 65years | Lepodisiran | placebo  Lepodisiran 4mg  Lepodisiran 12mg  Lepodisiran 32mg  Lepodisiran 96mg  Lepodisiran 304mg  Lepodisiran 608mg | -Adults between 18 to 65 years in USA and 21- to 65-year-old in Singapore  -adults without any known cardiovascular diseases  -BMI between 18.5 to 40  -Elevated lipoprotein(a) level (either≥75nmol/L or ≥30mg/dL). | - females of childbearing potential  -Consumed an excess amount of alcohol  -Smoked more than 10 cigarettes per day  -had a supine systolic/diastolic blood pressure that was greater than 160/90mmHg  -met criteria related to liver disease or kidney dysfunction | -Lipoprotein(a) | --- | Lepodisiran was well tolerated and produced dose-dependent, long-duration reduction in serum lipoprotein(a) concentrations |

**Supplementary table 5: Characteristics of the included ongoing studies.**

| Study ID | country | Study design | Study start date | Study completion  (estimated) | Sample size | Study intervention | Primary outcome | Secondary outcome | Inclusion criteria | Exclusion criteria |
| --- | --- | --- | --- | --- | --- | --- | --- | --- | --- | --- |
| NCT05537571 | Australia | Multi-center, Randomized, Double-blind, Placebo-controlled, Phase 2 Study | 3-01-2023 | 06-2024 | 160 | Drug: SLN360  Drug: Placebo | Time averaged change in Lp(a) from Baseline | -Lipoprotein(a)  -LDL-C  -Apolipoprotein (B) | -Lipoprotein(a) at screening equal to or greater than 125 nmol/L  -At high risk of ASCVD events  -A body mass index at screening in the range of 18.0 to 32.0 kg/m2 | -Renal dysfunction with estimated glomerular filtration rate less than 30 mL/min/1.73 m2 at screening |
| NCT04270760 | United states | Double-blind, Randomized, Placebo-controlled Phase 2 Study | 28-07-2020 | 08-11-2022 | 290 | Drug: Olpasiran  Drug: Placebo | Percent change in Lp(a) | -Lp(a)  -LDL-c  -Apo(B)  -C MAX of (Olpasiran)  -AUC of (Olpasiran) | -Age 18 to 80 years  -Lipoprotein (a) > 150 nmol/L  -Evidence of atherosclerotic cardiovascular disease | -Severe renal dysfunction  -History of hepatic dysfunction |
| NCT05581303 | United states | A Double-blind, Randomized, Placebo-controlled, Multicenter Study | 14-12-2022 | 16-12-2026 | 6000 | Drug: Placebo  Drug: Olpasiran | -Time to CHD death, myocardial infarction, or urgent coronary revascularization | -Percent change from baseline to Week 48 in Lipoprotein(a) | -Age 18 to ≤ 85 years  -Lp(a)≥ 200 nmol/L during screening  -History of ASCVD | -Severe renal dysfunction  -(AST) or (ALT) > 3 x ULN, or total bilirubin (TBL) > 2 x ULN during screening  -History of hemorrhagic stroke or major bleeding  - cardiac surgery or arterial revascularization  -Severe heart failure  -Current, recent, or planned lipoprotein apheresis  -received ribonucleic acid therapy target lipoprotein(a) |
| NCT04765657 | China | A Multicenter, Randomized, Double-blind, Parallel Group, Placebo-controlled Study | 01-03-2021 | 28-12-2026 | 345 | Drug: inclisiran sodium  Drug: Placebo | -LDL-C | -LDL-C  -PCSK9  -Apolipoprotein (B)  -LP(a)  -HDL-C  -non-HDL-C  -Triglycerides | At screening participants with: ASCVD and Serum LDL-C ≥1.8 mmol/L  Or ASCVD high and risk Serum LDL-C ≥2.6 mmol/L**.** | - NYHA class IV heart failure  - Cardiac arrhythmia or Major adverse cardiovascular event within 3 months prior to randomization.  - Uncontrolled severe hypertension  -history of malignancy |
| NCT05888103 | China | Randomized, Double-blind, Placebo-controlled Study | 11-07-2023 | 16-02-2025 | 200 | Drug: Inclisiran  Drug: Matching Placebo for Inclisiran | -LDL-C | -LDL-C  -PCSK9  -Apolipoprotein (B)  -Lipoprotein(a)  HDL-C  -non-HDL-C  -Triglycerides  -total cholesterol | -Written informed consent before any assessment  -Fasting LDL-C of ≥ 130 mg/dL but < 190 mg/dL  -Triglycerides ≤ 400 mg/dL  -low or moderate ASCVD risk by the 2016 Chinese Guideline | -History of ASCVD  -Diabetes mellitus or fasting plasma glucose of ≥ 7.0 mmol/L or HbA1c ≥ 6.5%  -Secondary hypercholesterolemia |
| NCT04987320 | Hong Kong | Open Label, Parallel, Randomized control trial | 28-07-2021 | 18-03-2022 | 24 | Drug: Olpasiran | (Cmax), (AUC), (Tmax), (t1/2), (Vz/F), (CL/F) of Olpasiran | -Treatment-emergent Adverse Events  -Change from Baseline in Serum Lipoprotein(a) | -participants with Lp(a) ≥ 70 nmol/L  -18 Years to 60 Years  -BMI between 18 and 32 kg/m^2  - no change in medication or dose during the study  - Females must be of non-reproductive potential | -history or evidence of peripheral neuropathy  -receiving apheresis  -history or evidence of bleeding diathesis or any coagulation disorder  -history or evidence of diabetes mellitus  -any herbal medicine, vitamins or dietary supplement affect lipid metabolism. |
| NCT04652726 | United States | Double-blind, Open label, Randomized Multicenter Study | 27-01-2021 | 03-12-2024 | 141 | Drug: Inclisiran  Drug: Placebo | Percent change of LDL-C | -LDL-c  -apolipoprotein (B)  -lipoprotein(a)  -non-HDL-C  -PCSK9 | -Heterozygous Familial Hypercholesterolemia  - LDL-C >130 mg/dL  -triglycerides <400 mg/dL  -(eGFR) >30 mL/min/1.73 m2 | -Homozygous familial hypercholesterolemia  -Active liver disease  -Secondary hypercholesterolemia  -treatment with monoclonal antibodies for PCSK9  -Major adverse cardiovascular events |
| NCT04659863 | United States | Double-blind, Open label, Randomized Multicenter Study | 16-02-2021 | 30-11-2024 | 13 | Drug: Inclisiran  Drug: Placebo | -LDL-C | -LDL-C  -apolipoprotein B  -lipoprotein (a)  -non-HDL-C  -HDL-C  -total cholesterol  -triglyceride | -Heterozygous Familial Hypercholesterolemia  - LDL-C >130 mg/dL  -triglycerides <400 mg/dL  -(eGFR) >30 mL/min/1.73 m2 | -Treatment with Mipomersen or Lomitapide  -Homozygous familial hypercholesterolemia  -Active liver disease  -Secondary hypercholesterolemia  -treatment with monoclonal antibodies for PCSK9  -Major adverse cardiovascular events |
| NCT04606602 | United States | Randomized, Double-blind, Placebo Controlled, First-in-human Study | 18-11-2020 | 08-2023 | 88 | Drug: SLN360  Drug: Placebo | -treatment-emergent adverse events | -(Cmax), (AUC), (CL/F) of SLN360  Pharmacodynamic: Change in Lp(a) | -Elevated plasma Lp(a) ≥ 150nmol/L  -BMI of ≥ 18 kg/m2 and ≤ 45 kg/m2.  -history of stable atherosclerotic cardiovascular disease | -history of clinically overt cardiovascular disease  - history of acute cardiovascular disease events within 6 months of screening  -Moderate or severe hepatic cirrhosis  -Active serious mental illness  -use therapies significantly affecting lipoprotein(a) level  -evidence of alcohol or illegal drug use within the 6 months before screening.  -drug allergies to an oligonucleotide |
| EUCTR2020-002757-18-SI | Brazil | Double-blind, Open label, Randomized Multicenter Study | 06/01/2021 | --- | 150 | Drug: Inclisiran  Drug: Placebo | Percent change of LDL-C | -LDL-c  -apolipoprotein (B)  -lipoprotein(a)  -non-HDL-C  -total cholesterol | Heterozygous Familial Hypercholesterolemia  - LDL-C >130 mg/dL  -triglycerides <400 mg/dL  -(eGFR) >30 mL/min/1.73 m2 | Homozygous familial hypercholesterolemia  -Active liver disease  -Secondary hypercholesterolemia  -treatment with monoclonal antibodies for PCSK9  -Major adverse cardiovascular events |
| LBCTR2021034776 | Australia | Double-blind, Open label, Randomized Multicenter Study | 31/08/2021 | 19/11/2022 | 4 | Drug: Inclisiran  Drug: Placebo | Percent change of LDL-C | -LDL-c  -apolipoprotein (B)  -lipoprotein(a)  -non-HDL-C  -total cholesterol  -PCSK9 | Heterozygous Familial Hypercholesterolemia  - LDL-C >130 mg/dL  -triglycerides <400 mg/dL  -(eGFR) >30 mL/min/1.73 m2 | Homozygous familial hypercholesterolemia  -Active liver disease  -Secondary hypercholesterolemia  -treatment with monoclonal antibodies for PCSK9  -Major adverse cardiovascular events |

Abbreviations: N, Number; mg/dL, Milligrams per Deciliter; nmol/L, Nanomoles per Liter; PCSK9, Proprotein Convertase Subtilisin/Kexin Type 9; IQR, Interquartile Range; ALT, Alanine Aminotransferase; AST, Aspartate Aminotransferase; CK, Creatine Kinase; ULN, Upper Limit of Normal; BMI, Body Mass Index; LDL-C, Low-Density Lipoprotein Cholesterol; HDL-C, High-Density Lipoprotein Cholesterol; DM, Diabetes Mellitus; NYHA, New York Heart Association; ASCVD, Atherosclerotic Cardiovascular Disease; eGFR, Estimated Glomerular Filtration Rate; Lp(a), Lipoprotein(a); Cmax, Maximum Observed Concentration; AUC, Area Under the Concentration-Time Curve; Tmax, Time to Maximum Observed Concentration; T1/2, Half-Life; Vz/F, Apparent Volume of Distribution During the Terminal Elimination Phase; CL/F, Apparent Total Body Clearance.

| **Supplementary table 6: Cochrane Risk of Bias Tool for Randomized Controlled Trials (ROB)** | | | | | | |
| --- | --- | --- | --- | --- | --- | --- |
| **References** | **Randomization process** | **Deviations from Intended Intervention** | **Missing outcome data** | **Measurement of the outcome** | **Selection of the reported result** | **Overall** |
| **Nissen et.al,2023** | **+** | **+** | **+** | **+** | **+** | low |
| **Donoghue et.al,2022** | **+** | **+** | **+** | **+** | **+** | low |
| **Koeing et.al,2022** | * | + | + | + | + | Some concerns |
| **Sohn et.al,2022** | - | + | + | + | + | high |
| **Nissen et.al 2022** | + | + | + | + | + | low |
| **Ray et.al 2022** | + | + | + | + | + | low |
| **Ray et.al 2020** | + | + | + | + | + | low |
| **Raal et.al2020** | + | + | + | + | + | low |
| **Fitzgerald et.al 2017** | - | * | + | + | + | high |

Cochrane Risk of Bias Tool for Randomized Controlled Trials (ROB)

(+): Yes/Low risk, (-): No/High risk, (*): inapplicable

**Supplementary table 7: Detailed search strategy**

| Central line through PubMed | | | |
| --- | --- | --- | --- |
| Domain searched | Serial | Search strategy | Results |
| [Title/Abstract] | #1 | ("short interfering RNA" OR "Small Interfering RNA" OR Oligonucleotides OR siRNA OR "Hairpin RNA" OR shRNA OR "Scan RNA" OR scnRNA OR tasiRNA OR "silencing RNA" OR RNAi OR esiRNA OR Patisiran OR ALN-18328 OR Onpattro OR Givosiran OR Givlaari OR Lumasiran OR Oxlumo OR ALN-GO1 OR Inclisiran OR Leqvio OR ALN-PCSsc OR ALN-60212 OR Pelacarsen OR Olpasiran OR SLN360) |  |
| [Title/Abstract] | #2 | ("Lipoprotein a" OR "Lipoprotein(a)" OR Lipoprotein-a OR Lipoproteina "Lp(a)" OR LPa OR "LP a" OR Lp-a OR Apo-A OR ApoA OR "Apolipoprotein A" OR "Apolipoprotein(a)" OR Apolipoprotein-a OR Apolipoproteina OR AK38 OR APOA) |  |
| [Title/Abstract] | #3 | #1 AND #2 | 507 |

| Scopus | | | |
| --- | --- | --- | --- |
| Domain searched | Serial | Search strategy | Results |
| Article Title, Abstract and Keywords | #1 | TITLE-ABS-KEY(("short interfering RNA" OR "Small Interfering RNA" OR Oligonucleotides OR siRNA OR "Hairpin RNA" OR shRNA OR "Scan RNA" OR scnRNA OR tasiRNA OR "silencing RNA" OR RNAi OR esiRNA OR Patisiran OR ALN-18328 OR Onpattro OR Givosiran OR Givlaari OR Lumasiran OR Oxlumo OR ALN-GO1 OR Inclisiran OR Leqvio OR ALN-PCSsc OR ALN-60212 OR Pelacarsen OR Olpasiran OR SLN360) AND ("Lipoprotein a" OR "Lipoprotein(a)" OR Lipoprotein-a OR Lipoproteina "Lp(a)" OR LPa OR "LP a" OR Lp-a OR Apo-A OR ApoA OR "Apolipoprotein A" OR "Apolipoprotein(a)" OR Apolipoprotein-a OR Apolipoproteina OR AK38 OR APOA)) | 251 |

| WOS | | | |
| --- | --- | --- | --- |
| Domain searched | Serial | Search strategy | Results |
| Topic | #1 | ("short interfering RNA" OR "Small Interfering RNA" OR Oligonucleotides OR siRNA OR "Hairpin RNA" OR shRNA OR "Scan RNA" OR scnRNA OR tasiRNA OR "silencing RNA" OR RNAi OR esiRNA OR Patisiran OR ALN-18328 OR Onpattro OR Givosiran OR Givlaari OR Lumasiran OR Oxlumo OR ALN-GO1 OR Inclisiran OR Leqvio OR ALN-PCSsc OR ALN-60212 OR Pelacarsen OR Olpasiran OR SLN360) AND ("Lipoprotein a" OR "Lipoprotein(a)" OR Lipoprotein-a OR Lipoproteina "Lp(a)" OR LPa OR "LP a" OR Lp-a OR Apo-A OR ApoA OR "Apolipoprotein A" OR "Apolipoprotein(a)" OR Apolipoprotein-a OR Apolipoproteina OR AK38 OR APOA) | 757 |

| Cochrane | | | |
| --- | --- | --- | --- |
| Domain searched | Serial | Search strategy | Results |
| Article Title, Abstract and Keywords | #1 | ("short interfering RNA" OR "Small Interfering RNA" OR Oligonucleotides OR siRNA OR "Hairpin RNA" OR shRNA OR "Scan RNA" OR scnRNA OR tasiRNA OR "silencing RNA" OR RNAi OR esiRNA OR Patisiran OR ALN-18328 OR Onpattro OR Givosiran OR Givlaari OR Lumasiran OR Oxlumo OR ALN-GO1 OR Inclisiran OR Leqvio OR ALN-PCSsc OR ALN-60212 OR Pelacarsen OR Olpasiran OR SLN360) AND ("Lipoprotein a" OR "Lipoprotein(a)" OR Lipoprotein-a OR Lipoproteina "Lp(a)" OR LPa OR "LP a" OR Lp-a OR Apo-A OR ApoA OR "Apolipoprotein A" OR "Apolipoprotein(a)" OR Apolipoprotein-a OR Apolipoproteina OR AK38 OR APOA) | 69 |

| Embase | | | |
| --- | --- | --- | --- |
| Domain searched | Serial | Search strategy | Results |
| Article Title, Abstract and Keywords | #1 | ("short interfering RNA" OR "Small Interfering RNA" OR Oligonucleotides OR siRNA OR "Hairpin RNA" OR shRNA OR "Scan RNA" OR scnRNA OR tasiRNA OR "silencing RNA" OR RNAi OR esiRNA OR Patisiran OR ALN-18328 OR Onpattro OR Givosiran OR Givlaari OR Lumasiran OR Oxlumo OR ALN-GO1 OR Inclisiran OR Leqvio OR ALN-PCSsc OR ALN-60212 OR Pelacarsen OR Olpasiran OR SLN360) AND ("Lipoprotein a" OR "Lipoprotein(a)" OR Lipoprotein-a OR Lipoproteina "Lp(a)" OR LPa OR "LP a" OR Lp-a OR Apo-A OR ApoA OR "Apolipoprotein A" OR "Apolipoprotein(a)" OR Apolipoprotein-a OR Apolipoproteina OR AK38 OR APOA) | 2974 |
